# Cryo-EM reveals structural variability of apolipoprotein A-I amyloid fibrils across organs, mutations, and clinical presentations

**DOI:** 10.1038/s41467-026-72150-z

**Published:** 2026-04-17

**Authors:** Binh An Nguyen, Maria del Carmen Fernandez-Ramirez, Parker Bassett, Virender Singh, Preeti Singh, Maja Pękała, Layla Villalon, Yasmin Ahmed, Andrew Lemoff, Bret Evers, Christian Lopez, Barbara Kluve-Beckerman, Lorena Saelices

**Affiliations:** 1 Center for Alzheimer’s and Neurodegenerative Diseases, Department of Biophysics, Peter O’Donnell Jr Brain Institute, University of Texas Southwestern Medical Center (UTSW), Dallas, TX USA; 2 SciKonnect and BioPatriKa, Naraingarh, Ambala, Haryana India; 3 Department of Biochemistry, University of Texas Southwestern Medical Center, Dallas, TX USA; 4 Department of Pathology, University of Texas Southwestern Medical Center (UTSW), Dallas, TX USA; 5 Department of Ophthalmology, University of Texas Southwestern Medical Center (UTSW), Dallas, TX USA; 6 Department of Internal Medicine, University of Texas Southwestern Medical Center (UTSW), Dallas, TX USA; 7 Department of Pathology and Laboratory Medicine, Indiana University School of Medicine, Indianapolis, IN USA

**Keywords:** Molecular biophysics, Cryoelectron microscopy, Protein aggregation

## Abstract

Hereditary apolipoprotein A-I (AApoA‑I) amyloidosis is a rare systemic disease caused by the deposition of amyloid fibrils formed by apolipoprotein A‑I in multiple organs, leading to severe clinical outcomes. With no available therapies or diagnostic tools, defining the structure of AApoA‑I fibrils is crucial to understanding disease mechanisms and guiding intervention. Here we use cryo-electron microscopy to analyze AApoA‑I fibrils from the heart, kidney, liver, and spleen of patients carrying G26R, L90P, and R173P mutations. G26R fibrils, regardless of organ, exhibits untwisted morphologies and cannot be resolved structurally. Conversely, L90P and R173P fibrils display a compact diabolo-shaped conformation in all organs analyzed. Their high-resolution maps enable visualization of *cis*-Proline 66, which may represent a potential conformational switch during fibril formation. Our findings suggest that mutation-driven polymorphism may influence organ tropism and clinical presentation. This work advances our understanding of AApoA‑I fibril assembly and provides insights toward developing targeted clinical tools.

## Abstract (web summary)

Cryo-EM analysis of amyloid fibrils from patients with hereditary ApoA-I amyloidosis shows that different disease mutations can produce distinct fibril structures depending on their position relative to the fibril core, which may influence organ damage and clinical outcomes.

## Introduction

Hereditary apolipoprotein A-I amyloidosis (AApoA-I amyloidosis) is a rare systemic disease caused by the deposition of amyloid fibrils composed of apolipoprotein A-I (ApoA-I). These fibrils accumulate in multiple organs, including the liver, kidneys, heart, peripheral nerves, gastrointestinal tract, skin, eyes and testes, leading to tissue damage, organ failure, and eventually death^1^. Disease onset varies widely, with symptoms often arising between the ages of 20 and 70^2^. Standard amyloid testing, such as Congo Red or thioflavin S staining, does not differentiate AApoA-I amyloidosis from other amyloid types without genetic or proteomic analysis, often resulting in misdiagnosis and suboptimal treatment^3^. Current treatment is primarily supportive, with liver transplantation as the main option to replace mutated ApoA-I with a wild-type ApoA-I, or organ transplantation to address failing or damaged organs^4^. Similar to other rare amyloidosis diseases, a major challenge in the field of AApoA-I amyloidosis is the limited understanding of disease mechanisms leading to variability of clinical presentations.

ApoA-I is a multifunctional protein with a well-defined structural organization that plays a central role in lipid metabolism and cardiovascular protection. The matured ApoA-I protein consists of 243 residues primarily synthesized in the liver and the intestines^5^. Its native structure is predominantly amphipathic α-helices making up of ten tandem repeats that allow it to interact with phospholipids effectively^6–8^. ApoA-I has three functional domains that contribute to its functions in lipid metabolism. An N-terminal domain (residues 1–184) stabilizes the lipid-free ApoA-I, facilitates binding to lipid due to its amphipathic α-helix structure, and enables interactions with lipids to form high-density lipoproteins (HDLs)^9^. Overlapping with this domain, a central region (residues 144–164) is crucial for activating lecithin-cholesterol acyltransferase, an enzyme essential for cholesterol esterification within HDL particles, and for cellular cholesterol efflux^10–12^. Finally, a C-terminal domain (residues 185–243) allows ApoA-I to anchor to lipid surfaces, increasing its binding capacity^13,14^. Functionally, wild-type ApoA-I is a crucial component of HDLs, which are responsible for removing excess cholesterol from peripheral tissues and transporting it to the liver for excretion. This cardiovascular protective mechanism, known as reverse cholesterol transport, helps mitigate atherosclerosis by reducing lipid buildup in blood vessel walls and maintaining cholesterol balance in the body^10,13^.

Despite its protective role, both wild-type and variant ApoA-I can misfold and aggregate under certain conditions. To date, over 20 pathogenic mutations in ApoA-I have been identified, with many clustering in two residue stretches: residues 26–75 and 154–178^6,15^. Proteomic analysis of ex vivo AApoA-I amyloid fibrils has consistently shown that the fibril core is primarily composed of N-terminal fragments, typically covering the first 83–98 residues, regardless of mutation sites^16–18^. The current evidence suggests that mutations destabilize the native conformation of ApoA-I, exposing the N-terminal aggregation-prone regions (APRs) and initiating amyloid formation^19–21^. Yet, the precise molecular mechanism linking mutations to fibril formation, and how these changes translate to clinical outcomes, remains poorly understood.

Previous studies have reported that the position of the ApoA-I mutation correlates with distinct patterns of tissue deposition and subsequent clinical manifestations^22,23^. Specifically, mutations between residues 26 and 75 are associated with deposition in the kidney and liver, while those between residues 90 and 178 tend to localize in the heart, larynx, and skin (Supplementary Table 1)^22–24^. Additionally, amyloid deposition of wild-type ApoA-I has been observed in the arterial walls of individuals with atherosclerosis^25^. The mechanism by which ApoA-I variant position drives organ tropism remains poorly understood. In neurodegenerative amyloid diseases, such as tauopathies or synucleinopathies, each clinical syndrome is associated with a defined structure (or structures) of the amyloid fibrils^26–28^. Inspired by these studies, we hypothesize that the clinical presentations in AApoA-I amyloidosis may be linked to the structural conformation of the amyloid fibrils, which could, in turn, be determined by the site of the mutation.

In this study, we determined the structures of ex vivo AApoA-I amyloid fibrils extracted from multiple organs and patients. Samples included fibrils extracted from heart, liver, kidney, and/or spleen from three patients: one patient with renal and hepatic involvement carrying the G26R mutation, and two with cardiac and cutaneous manifestations carrying the L90P and R173P mutations^29–31^. Mass spectrometry (MS) and cryogenic electron microscopy (cryo-EM) revealed distinct fibril conformations associated with different clinical phenotypes, while structural consistency was observed across tissues within the same patient. Our preliminary data suggest that the mutation site may influence fibril architecture, potentially contributing to phenotypic heterogeneity.

## Results

### Histological study of amyloid deposits

**Fig. 1:**
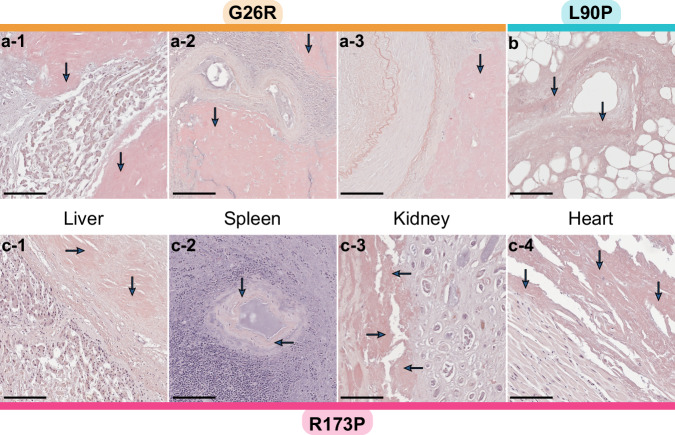
Histological analysis of AApoA-I amyloid deposits of various tissue samples from three patients, G26R, L90P and R173P, using Congo red staining. Arrows indicate amyloid deposits. Cross-sections show amyloid in the liver, spleen and kidney of G26R (**a**1–3), the heart of L90P (**b**), and the liver, spleen, kidney and heart of R173P (**c**1–4). Scale bar: 150 µm.

**Fig. 2:**
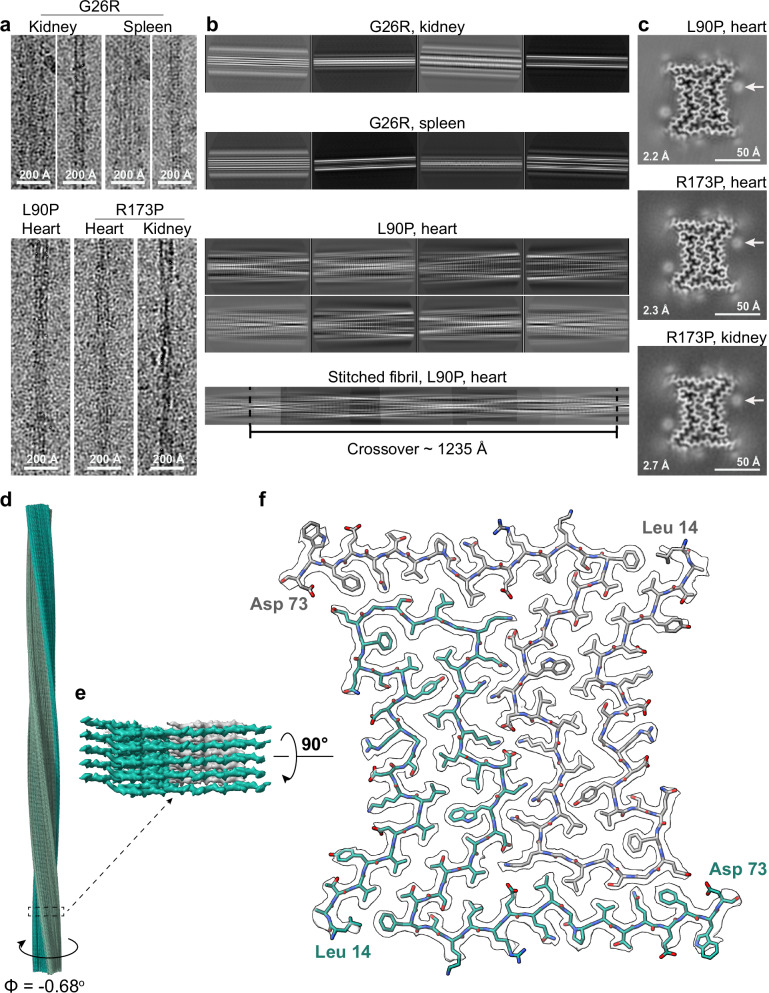
Cryo-EM images of AApoA-I fibrils, their 2D and 3D classifications, and the reconstructed fibril from L90P variant. **a** Cryo-EM images of fibrils from G26R kidney and spleen, L90P heart and R173P heart and kidney. Scale bar: 200 Å. **b** 2D class averages (particle box size of 300 pixels) of G26R fibrils from the kidney and spleen, and L90P heart. Stitched 2D class averages of fibrils showing the crossover from L90P variant. **c** Cross sections through the reconstructed L90P, R173P heart and kidney fibrils, perpendicular to the helical axis. Resolutions in Å are indicated on the bottom left. White arrows point to the extra densities. Scale bar: 50 Å. **d** The reconstructed L90P fibril containing 360 layers. **e** Magnified view of (**d**) showing a subset of five fibril layers. **f** Cryo-EM map and atomic model of AApoA-I fibrils are shown for a single layer perpendicular to fibril axis.

**Fig. 3:**
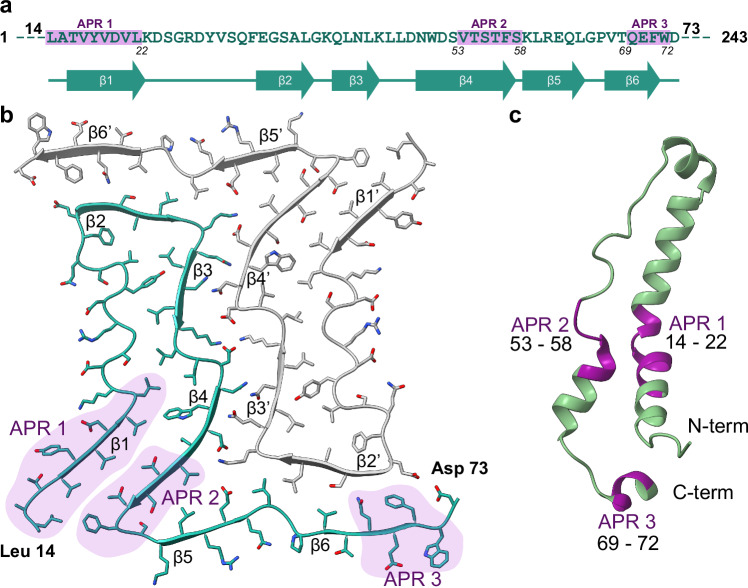
Structural information of AApoA-I fibrils and the location of aggregation-prone regions. **a** Amino acid sequence (green) of the AApoA-I fibril core; the regions highlighted in purple are the APRs; green arrows indicate β-strands. **b** Top view of a single fibril layer from L90P variant showing the arrangement of β-strands and location of APR1 to APR3 (in purple area). **c** Native crystal structure of ApoA-I spanning residues 2–75 (PDB: 3R2P) and the locations of APRs are highlighted in purple.

We obtained freshly frozen samples from three patients with hereditary AApoA-I amyloidosis and conducted histological analysis using Congo red, Thioflavin S, and hematoxylin and eosin stains (Fig. 1 and Supplementary Fig. 1a, b). Due to sample availability, we histologically analyzed only select organs for each patient. However, we identified amyloid deposits in all tissues available for analysis, though the amount of fibril deposition varied. Patient 1 (G26R), who died of renal failure, carried the G26R mutation^29^. We observed amyloid deposits in the liver (among hepatocytes, Fig. 1a-1), spleen (in red pulp regions, Fig. 1a-2) and kidney (in the medulla, Fig. 1a-3). Patient 2 (L90P), who died of heart failure and presented cutaneous lesions, carried the L90P mutation^31^. We observed amyloid deposits around the epicardial coronary artery of the heart (Fig. 1b). Patient 3 (R173P), who died of heart, renal, and liver failure, carried the R173P mutation^30^. We observed amyloid deposits in the liver (among hepatocytes, Fig. 1c-1), spleen (surrounding the central artery, Fig. 1c-2), kidney (in the hilar fascia, Fig. 1c-3), and heart (in the extracellular space of myocardial cells, Fig. 1c-4).

### Fibril extraction and protein identification by MS

We purified AApoA-I fibrils from all samples using an ice-cold water extraction protocol, as previously described^32^. Transmission electron microscopy (TEM) of the resulting eluates confirmed the presence of amyloid fibrils in all tissue samples (Supplementary Fig. 2), consistent with histological staining. We confirmed the ApoA1 identity of these isolated ex vivo fibrils using MS. Tryptic analysis revealed ApoA-I sequence coverage ranging from 71 to 89% across all fibril extracts (Supplementary Fig. 3a and Supplementary Table 2), together with the detection of established amyloid signature proteins such as serum amyloid-P component, apolipoprotein E and apolipoprotein A-IV. Using intact MS, we identified a fragment spanning residues 1–81 as the predominant species across all tissues and patients (Supplementary Figs. 3b and  4). Additional patient-specific fragments were also identified: fragments spanning residues 1–67 and 1–71 in all G26R samples, 1–85 and 1–93 in all L90P samples, and 1–85 in all R173P samples (Supplementary Fig. 4). We confirmed heterozygosity in all patients, consistent with previously reported. Tryptic digestion was used in most cases; however, GluC digestion was employed to detect the R173P mutation, as tryptic cleavage around the mutation generated peptide fragments that are below the reliable MS detention threshold.

### Cryo-EM reveals structural heterogeneity of G26R fibrils vs both L90P and R173P fibrils

We prepared cryo-EM grids using elution fractions that contained high-quality fibrils, based on fibril concentration and morphology assessed by TEM (Supplementary Fig. 2). We excluded three extracts—the G26R liver sample and the R173P liver and spleen samples—because of excessive fibril bundling or low yield. The remaining fibril extracts showed well-dispersed, abundant fibrils, which enabled cryo-EM structure determination. These included fibrils from the kidney and spleen of the G26R patient, the heart of the L90P patient, and the heart and kidney of the R173P patient.

Two-dimensional (2D) class averages provided initial insight into the structural characteristics of fibrils across patient samples. (Fig. 2a). Renal and splenic fibrils from the G26R patient displayed diameters of ~7.5–19 nm and lacked apparent twist, rendering them unsuitable for helical reconstruction (Fig. 2b). In contrast, cardiac fibrils from the L90P and R173P patients, as well as renal fibrils from the R173P patient, exhibited twisted morphology with diameters of ~9.5 nm (Fig. 2b and Supplementary Figs. 5 and 6a, b). We used the resulting 2D classes from twisted fibrils to generate initial models for further structural processing.

We determined the structures of the ordered cores of AApoA-I fibrils from the L90P heart, the R173P heart, and the R173P kidney using helical reconstruction of cryo-EM images, achieving resolutions of 2.2 Å, 2.3 Å, and 2.7 Å, respectively (Fig. 2c and Supplementary Fig. 7). The fibril folds were identical across all samples, regardless of tissue origin or mutation, indicating structural conservation. Each fibril was composed of two intertwined protofilaments forming a compact, diabolo-shaped core (Fig. 2c). The fibril layers were separated by a rise of 4.85 Å and a twist angle of −0.67° ( ± 0.01) (Fig. 2d, e and Supplementary Fig. 6c, d). We also observed two unidentified round densities ( ~ 8 Å in diameter) positioned outside the fibril core, along with weaker densities at the fibril ends, suggesting the presence of putative binding molecules and/or disordered regions at both the N- and C-termini of ApoA-I (white arrows in Fig. 2c).

The high resolution of the AApoA-I fibril density maps enabled us to determine the handedness of the helical twist and to build accurate atomic models (Supplementary Fig. 8a). The ordered core of each protofilament spanned residues Leu 14 or Ala 15 to Asp 73 or Trp 72, placing the mutations outside the structured region (Fig. 2f and Supplementary Fig. 6e, f). The alignment of the Cα backbones from the three fibril structures—L90P heart, R173P heart, and R173P kidney—revealed high structural homogeneity, with an average root mean square deviation (r.m.s.d.) of 0.15 Å, as calculated by GESAMT (Supplementary Fig. 8b, c). The MS analysis of fibril extracts supports this model, identifying two major cleavage-prone regions flanking the AApoA-I fibril core in all variants (Supplementary Fig. 9).

### Analysis of the L90P and R173P fibril structures

AApoA-I fibrils rank among the highly stable amyloid assemblies characterized to date^33^. In silico structural analysis estimates their solvation energies at ~66.6 kcal/mol per layer, −33.3 kcal/mol per molecule, and −0.56 kcal/mol per residue (Supplementary Fig. 10a). This exceptional stability arises from a dense interaction network that reinforces the protofilament core and supports interprotofilament and interlayer contacts.

Hydrophobic interactions stabilize the protofilament core, while polar interactions maintain the interface between protofilaments. Each AApoA-I molecule contributes six β-strands, plus a loop between Leu22 and Phe33 (Fig. 3a). The first four β-strands form a long hydrophobic hairpin, and β6 interacts with the β2′ strand from the neighboring protofilament (Fig. 3b; prime indicates the adjacent protofilament). The two protofilaments also interact through salt bridges and hydrogen bonds, as shown in Supplementary Fig. 10b and Supplementary Fig. 11a, b. Additionally, we observe salt bridges on the solvent-exposed surface (Supplementary Fig. 11c), typical π–π interactions of aromatic residues, hydrogen bonding from stacked aspartate and glutamate side chains and an interlayer hydrogen bonding network formed by the peptide backbone—all characteristic features of the amyloid architecture (Supplementary Fig. 11d–f). Notably, Proline 66 (*n*) adopts a cis-isomer conformation in both protofilaments, enabling the formation of a hydrogen bond with Gln 63 from the layer below (*n*−1) (Supplementary Fig. 11g). Finally, we observe small densities embedded at the protofilament interface within the fibril, likely water molecules that form hydrogen bonding along the fibril axis (Supplementary Fig. 11h).

### Analysis of APRs of AApoA-I

In silico analysis of the AApoA-I fibril structure revealed key APRs that potentially drive and stabilize fibril assembly through steric zippers and aromatic interactions. Four key APRs were identified; three located within the AApoA-I fibril, corresponding to β1 (APR1), β4 (APR2) and β6 (APR3) strands (Fig. 3). The β1 and β4 strands form an extended N-to-C hetero-steric zipper that brings APR1 and APR2 together, maintaining a similar proximity as seen in the ApoA-I crystal structure (Fig. 3b, c)^34^. Additionally, the β4 strand establishes a shorter N-to-C zipper on the opposite side, involving interactions between Asn 49 and Asp 51 with Asn 43’ and Gln 41’ on the β3’ strand. The β6 strand formed an N-to-C zipper with the β2’ strand from the adjacent protofilament. Interestingly, all identified APRs within the fibril core include at least one aromatic residue: Phe 33 in APR1, Tyr 50 in APR2, and Phe 71 and Trp 72 in APR3. A fourth APR, spanning residues Val 221–Leu 233 at the C-terminus, was predicted to lie outside the fibril core.

## Discussion

The clinical presentation of AApoA-I amyloidosis varies significantly, including affected organs, symptom severity, and age of onset. Symptoms often overlap with other diseases, complicating timely diagnosis and effective clinical management^4,35^. Previous studies have established associations between specific mutation sites and organ involvement, though a mechanism behind this phenomenon has yet to be deduced^24,36^. In this study, we present cryo-EM structures of AApoA-I fibrils isolated from multiple organs of three patients with distinct genotypes and clinical presentations. Our preliminary results suggest that mutations in the *ApoA-I* gene affect the structure of AApoA-I fibrils, which potentially contribute to the observed heterogeneity in disease manifestation.

The location of single-point mutations in ApoA-I has been associated with distinct clinical phenotypes. Studies have shown that mutations within the N-terminal region (residues 1–75) are predominantly linked to renal and hepatic pathologies, while mutations beyond residue 90 are more commonly associated with cardiac and laryngeal involvement, albeit not exclusively^15,36–38^ (Supplementary Table 1). Our structural analyses demonstrate that fibrils formed by the G26R variant adopt a distinct, untwisted morphology, whereas fibrils carrying L90P and R173P mutations exhibit a consistently twisted morphology (Fig. 2a, b). This morphological divergence suggests that mutation-specific alterations in fibril architecture may play a critical role in modulating tissue tropism and organ-specific amyloid deposition^22^. Comparable correlations between fibril structure and disease phenotype have been observed in tauopathies and synucleinopathies, neurodegenerative diseases characterized by tau and ɑ‑synuclein protein aggregation, respectively^26–28^. These diseases include Alzheimer’s, Parkinson’s, chronic traumatic encephalopathy, and Lewy Body dementia, amongst others. Structural studies in these diseases have shown that each clinical syndrome is associated with a unique filament fold, reinforcing the importance of fibril conformation in disease expression. Our analysis of AApoA-I fibrils supports a potential connection between untwisted fibril morphologies and renal/hepatic phenotypes, and twisted fibril structures and cardiac involvement. Further investigation of additional AApoA-I variants across diverse tissues will be critical to elucidate how mutation location influences fibril polymorphism, deposition site, and disease presentation.

To explore the structural basis underlying these phenotypic associations, we examined how mutation location may impact the integrity of the fibril core and, consequently, fibril morphology. Cryo-EM helical reconstruction of L90P and R173P fibrils revealed a conserved diabolo-shaped core composed of two intertwined protofilaments, consistent across multiple organs in these patients (Fig. 2f and Supplementary Fig. 6b). This fibril core, spanning residues 14–73, appears unaffected by mutations outside this region. In contrast, the G26R substitution would introduce a bulky, ionic residue directly into a hydrophobic pocket, likely incompatible with the proper folding of this fibril core and perhaps resulting in a distinct untwisted fibril morphology (Fig. 2b). Additionally, G26R fibrils displayed morphological heterogeneity (Fig. 2a, b). Given that the G26R patient was heterozygous, this variability may arise from different contributions of wild-type and variant molecules. Similar structural incompatibility may occur with other mutations located within the fibril core, such as W50R, L60R, L64P, and F71Y, due to potential steric clashes or interference with key hydrophobic interactions (Supplementary Fig. 12). These destabilizing effects could favor the emergence of alternative fibril morphologies such as the untwisted species observed in the G26R patient (Fig. 2a, b).

Mutations may also influence the proteolytic profile of ApoA-I and may be shaped by, or contribute to, the distinct fibril morphologies observed in our samples. In vitro studies have shown that mutations such as G26R and L178H increase susceptibility to N-terminal proteolysis compared to the wild-type protein, likely due to structural alterations in the lipid-binding C-terminal domain^18,39^. These changes diminish lipid-binding ability, rendering the protein more prone to proteolytic activities^40^. Notably, different mutations can affect the lipid-binding ability of ApoA-I to varying degrees, leading to divergent proteolytic outcomes^41^. This variability may explain our intact MS results, which revealed unique N-terminal fragments of different lengths among fibrils from distinct variants (Supplementary Fig. 9).

Proteolysis may play a dual role in ApoA-I fibril formation. Some studies propose that proteolysis precedes and facilitates fibril formation by exposing aggregation-prone N-terminal fragments^42,43^. In vitro studies demonstrate that full-length ApoA-I, both wild-type and G26R mutant, does not form fibrils under various conditions, whereas the N-terminal fragment (residues 1–83) of both constructs readily assembles into fibrils^43,44^. Alternatively, other studies propose that APRs of ApoA-I become exposed due to mutation-induced conformational changes, with fibril formation preceding proteolysis^19,45^. Another study proposes that full-length ApoA-I may form fibrils upon methionine oxidation in vitro^46^. Together, these findings raise the possibility that the unique truncated fragments observed in our study may arise through two scenarios: in one, specific proteolytic fragments seed distinct fibril morphologies; in the other, structural differences in fibrils expose additional protease cleavage sites. Our current data do not allow us to determine the sequence or causality of these events, leaving open questions about the complex relationship between mutation, proteolysis, and fibril formation in AApoA-I amyloidosis.

Our fibril structures offer insights into a potential mechanism of ApoA-I aggregation. While mutation location may dictate fibril structure and proteolysis may be a prerequisite for fibril formation, the exposure of APRs likely drives the process. The pronounced structural difference between the native and amyloid forms of ApoA-I suggests a conformational conversion involving destabilization of the native protein and reassembly into a β-sheet–rich architecture. In vitro studies reveal that mutations such as G26R and L178H destabilize the helical structure of full-length ApoA-I, potentially exposing APRs^18,39^. Notably, APR1 and APR2, although distant in sequence, are spatially adjacent in the ApoA-I crystal structure (Fig. 3c and Supplementary Fig. 13a) and converge to form a steric zipper in the fibril core (Fig. 3b and Supplementary Fig. 13b). In particular, the side chains from Val19 in APR1 and Thr56 in APR2 maintain proximity in both conformations (3.72 Å in the native, 4.31 Å in fibrils, Supplementary Fig. 13), suggesting that this contact may nucleate the zipper interface. Additionally, Thr54 may reorient inward, and together with Thr56, stabilize the APR1-APR2 steric zipper via coordination with two water molecules (Supplementary Figs. 11h and 13b). The critical role of APR1 and APR2 in fibril formation of ApoA-I was first demonstrated in vitro by Mizuguchi et al.^47^, who showed that eliminating APR1 (residues 14–22) inhibited fibril formation of the G26R fragment spanning residues 1–83, whereas eliminating APR2 (residues 50–58) significantly reduced nucleation and fibril growth rates.

Adding to this mechanism, our data reveal the presence of Pro66 in a cis-isomer conformation in both protofilaments of all determined structures (Supplementary Fig. 11g). Proline isomerization, a unique type of post-translational conformational change, is known to play an important role in amyloid formation and is proposed to act as a conformational switch that influences protein folding and refolding^48,49^. This conversion may occur either before (and potentially trigger) or after the disruption of ApoA-I’s α-helical secondary structure. In either case, the 180° flip of the Pro66 peptide bond would enable an additional hydrogen bond with Gln63 in the adjacent layer, further stabilizing the fibril core (Supplementary Fig. 11g). These findings underscore the importance of APR exposure and proline isomerization of the native ApoA-I structure in AApoA-I amyloidosis.

In summary, this study provides structural insights into ex vivo AApoA-I fibrils obtained from multiple organs of patients carrying distinct mutations with varying clinical manifestations. The observed fibril heterogeneity, possibly driven by mutation location, may play a key factor in tissue tropism and disease outcome in hereditary AApoA-I amyloidosis. While only the L90P and R173P fibrils were structurally characterized, the tentative conclusions regarding G26R fibrils emphasize the need for further structural investigation. Our proteolysis data and APR analysis align with previous findings, reinforcing the central role of APRs in fibril formation. Furthermore, the discovery of Pro66 in a cis-isomer conformation in the fibril structure, but not in native ApoA-I, highlights the potential contribution of a conformational switch to amyloidogenesis. While our conclusions are based on a limited number of patient-derived samples, the trends observed support the need for broader investigation to fully elucidate the interplay between mutation, fibril structure, and clinical presentation.

## Methods

### Ethical statement

These patient samples were fully anonymized prior to transfer and were therefore exempt from Institutional Review Board oversight by UT Southwestern Medical Center’s Office of the Human Research Protection Program. Informed consent for tissue collection and research use was obtained under the protocols governing the original studies conducted at Indiana University School of Medicine. No participants were recruited for the present study, and no compensation was provided.

### Patients and tissue materials

We obtained human tissue samples from the laboratory of Dr. Merrill D. Benson at the Indiana University School of Medicine. These specimens were postmortem frozen tissues from three patients (two females and one male, age range 60–65) diagnosed with AApoA-1 amyloidosis. Patient 1, carrying the G26R mutation, passed away from renal failure; the kidney, spleen, and liver tissues were used in this study^50^. Patient 2, with the L90P mutation, died from heart failure and also had cutaneous lesions. Heart tissue from this patient was used in this study^31^. Patient 3, with the R173P mutation, died with cardiomyopathy, liver, and renal failure; the heart, kidney, spleen, and liver were used in this study^30^. The remaining material is stored at our institution, but cannot be freely distributed due to ethical and regulatory restrictions.

### Histology

Fresh-frozen human cardiac tissue was thawed overnight at 4 °C and then fixed in 20x volume excess of 10% neutral buffered formalin for 48 hours at room temperature with agitation. After fixation, samples were transferred to 70% ethanol and paraffin processed according to established protocols^51^. Sections were generated from paraffin blocks for hematoxylin and eosin staining (H&E), Congo Red and Thioflavin-S staining. Regressive H&E was performed on a Sakura DRS-601 x,y,z robot utilizing Leica-Surgipath Selectech reagents according to Sheehan’s textbook methodology. Congo Red slides counterstained with hematoxylin were evaluated for pathologic amyloid aggregation under bright-field following their preparation. Color was imparted to amyloid by methods also described in Sheehan’s text utilizing 0.1% Congo Red in alcoholic-saline following sensitization of the slides with alkaline-alcoholic-saline (10% NaCl, 0.5% NaOH, and 80% EtOH). Thioflavin-S slides were evaluated for foci consistent with plaque amyloid and vascular amyloid, which fluoresce brilliantly when imaged with ultraviolet excitation (400–440 nm) and long-pass emission (470 nm + ). Thioflavin-S staining was accomplished utilizing methods described by Guntern et al.^52^. In brief, paraffin sections were brought to water and prepared for alcoholic Thioflavin-S impregnation by sequential oxidation with 0.25% w/v potassium permanganate, bleaching (1% potassium metabisulfite, 1% oxalic acid), peroxidation (1% hydrogen peroxide, 2% sodium hydroxide), and acidification (0.25% acetic acid), all with interceding water washes. Slides were then brought to 50% ethanol and incubated in alcoholic Thioflavin-S (0.004%). After seven minutes staining interval, slides were passed through alcoholic rinses to remove excess Thioflavin-S, dehydrated, cleared, and cover slips affixed with Cytoseal 60 permanent non-fluorescent mounting media (Epredia, Kalamazoo, MI).

### Extraction of AApoA-1 amyloid fibrils

AApoA-1 ex vivo preparation of amyloid fibrils was obtained from the fresh-frozen human tissue as described earlier, with slight modifications^32,53^. Briefly, ~250 mg of frozen tissue was thawed at room temperature and minced into small pieces with a scalpel. The minced tissue was resuspended into 0.5 mL Tris-calcium buffer (20 mM Tris, 138 mM NaCl, 2 mM CaCl_2_, 0.1% NaN_3_, pH 8.0) and centrifuged for 5 min at 7000 × *g* and 4 °C. The pellet was washed in Tris-calcium buffer four additional times. After washing, the pellet was resuspended in 0.5 mL of 5 mg/mL collagenase solution (*Clostridium histolyticum* collagenase type IV stock prepared in Tris-calcium buffer). Sample was spiked with a complete protease inhibitor cocktail EDTA-free tablets (Roche) and incubated overnight at 37 °C with shaking at 700 rpm (Eppendorf ThermoMixer). The resuspension was centrifuged for 30 min at 7000 × *g* at 4 °C, and the pellet was resuspended in 0.5 mL Tris–ethylenediaminetetraacetic acid (EDTA) buffer (20 mM Tris, 140 mM NaCl, 10 mM EDTA, 0.1% NaN_3_, pH 8.0). The suspension was centrifuged for 5 min at 7000 × *g* and 4 °C, and the washing step with Tris–EDTA was repeated nine additional times. After final washing, the pellet was resuspended in 150 μL ice-cold water supplemented with 5 mM EDTA. After a 15-minute incubation on ice, the sample was centrifuged for 5 min at 3100 × *g* and 4 °C. This extraction step was repeated three additional times, and elutions were collected for further analysis.

### Negative-stained TEM

The extracted AApoA-1 amyloid fibril was confirmed by TEM. A 2.5 μL aliquot of the fibril sample was spotted onto a freshly glow-discharged carbon film 300 mesh copper grid (Electron Microscopy Sciences) and incubated for 1 min. Excess solution was gently blotted with filter paper. The grid was then stained with 2.5 µL of 2% uranyl acetate for 1 min, followed by gently blotting to remove excess stain. A second application of 2.5 μL uranyl acetate was immediately blotted, and the grid was air-dried vertically for at least 2 min. Specimens were imaged using an FEI Tecnai 12 electron microscope (Thermo Fisher Scientific) at an accelerating voltage of 120 kV.

### MS sample preparation, data acquisition and analysis

For enzymatic MS analysis, 0.5 µg of extracted fibrils were dissolved in a tricine SDS sample buffer, boiled for 2 minutes at 85 °C, and run on a Novex™ 16% tris-tricine gel system using a Tricine SDS running buffer. Gel was stained with Coomassie dye, destained and the smear with AApoA-1 was cut from the gel. Sample was sent for MS analysis. Samples were digested overnight with trypsin (Pierce) following reduction and alkylation with DTT and iodoacetamide (Sigma–Aldrich). The samples then underwent solid-phase extraction cleanup with an Oasis HLB plate (Waters), and the resulting samples were injected into a Q Exactive HF mass spectrometer coupled to an Ultimate 3000 RSLC-Nano liquid chromatography system. Samples were dissolved in Buffer A (2% (v/v) ACN and 0.1% formic acid in water), injected onto a 75 µm i.d., 15-cm long EasySpray column (Thermo), and eluted with a gradient from 0–28% buffer B over 90 min. Buffer B contained 80% (v/v) ACN, 10% (v/v) trifluoroethanol, and 0.1% formic acid in water. The mass spectrometer operated in positive ion mode with a source voltage of 2.5 kV and an ion transfer tube temperature of 300 °C. MS scans were acquired at 120,000 resolution in the Orbitrap with a scan range of m/z = 350–1700, and up to 20 MS/MS spectra were obtained in the ion trap for each full spectrum acquired using higher-energy collisional dissociation (HCD) for ions with charges 2–8. Dynamic exclusion was set for 20 s after an ion was selected for fragmentation. For the identification of AApoA-1 R173P mutation, Glu-C enzyme (Promega) was used instead of trypsin, with no other steps in the protocol altered, as tryptic digestion in the R171-177 region generated peptide fragments below the reliable MS detection range.

Raw MS data files were analyzed using Proteome Discoverer v3.0 SP1 (Thermo), with peptide identification performed using a semitryptic (or semi-Glu-C for the R173P mutant sample) search with Sequest HT against the human reviewed protein database from UniProt, along with the G26R, l90P, and R173P ApoA-I mutant sequences. Fragment and precursor tolerances of 10 ppm and 0.02 Da were specified, and three missed cleavages were allowed. Carbamidomethylation of Cys was set as a fixed modification, with oxidation of Met set as a variable modification. The false-discovery rate cutoff was 1% for all peptides, and peptides needed to have at least two peptide spectral matches and an XCorr value >3.00 to be considered.

For Intact protein mass analysis, 20 µL ( ~ 5 µg) of extracted AApoA-1 fibrils were spun at 21,000 × *g* for 1 h at 4 °C. The 15 µL of supernatant was removed, and 5 µL of 8 M GuHCl was added to dissociate the fibrils. Mixture was incubated at 37 °C for 1 h with continuous shaking at 1000 rpm (Eppendorf ThermoMixer). Then, an additional 5 µL of 8 M GuHCl was added and incubated at 37 °C for 1 hour with continuous shaking at 1000 rpm. Finally, 5 µL of MilliQ water containing reducing agent (DTT) was added. The GnHCl treatment only partially disrupted AApoA-I fibrils, resulting in heterogeneous mixtures of residual fibrils, oligomers and monomeric species, which limited chromatographic resolution and precluded reliable detection of intact full-length ApoA-I. Samples were desalted and analyzed by LC/MS, using a Sciex X500B QTOF mass spectrometer coupled to an Agilent 1290 Infinity II HPLC. Samples were injected onto a POROS R1 reverse-phase column (2.1 × 30 mm, 20 µm particle size, 4000 Å pore size) and desalted. The mobile phase flow rate was 300 μL/min and the gradient was as follows: 0–3 min: 0% B, 3–4 min: 0–15% B, 4–16 min: 15–55% B, 16–16.1 min: 55–80% B, 16.1–18 min: 80% B. The column was then re-equilibrated at initial conditions prior to the subsequent injection. Buffer A contained 0.1% formic acid in water, and buffer B contained 0.1% formic acid in acetonitrile.

The mass spectrometer was controlled by SciexOS v.3.3.1.43 using the following settings: Ion source gas 1 30 psi, ion source gas 2 30 psi, curtain gas 35, CAD gas 7, temperature 300 °C, spray voltage 5500 V, declustering potential 135 V, collision energy 10 V. Data was acquired from 400 to 2000 Da with a 0.5 s accumulation time and four time bins summed. The acquired mass spectra for the proteins of interest were deconvoluted using Bio Tool Kit within SciexOS in order to obtain the molecular weights. Peaks were deconvoluted over the entire mass range of the mass spectra, with an output mass range of 7000–9000 Da, using a low input spectrum isotope resolution.

The MS data have been deposited to MassIVE data repository (a member of ProteomeXchange), with accession number MSV000098187.

### Cryo-EM sample preparation and data collection

Freshly extracted fibril samples were applied to glow-discharged R1.2/1.3, 300-mesh copper grids (Quantifoil), blotted with filter paper to remove excess sample, and plunge-frozen in liquid ethane using a Vitrobot Mark IV (FEI/Thermo Fisher Scientific). Cryo-EM samples were screened on Talos Arctica at the Cryo-Electron Microscopy Facility (CEMF) at The University of Texas Southwestern Medical Center (UTSW). Images were collected using a 300 keV Titan Krios microscope (FEI/Thermo Fisher Scientific) with Falcon4i detector operated at a slit width of 10 eV at the Stanford-SLAC Cryo-EM Center (S^2^C^2^). Further details are provided in Supplementary Table 2.

### Helical reconstruction

Summary of cryo-EM data processing workflow is illustrated in Supplementary Fig. 5. The raw movie frames were gain-corrected, aligned, motion-corrected and dose-weighted using RELION’s own implemented motion correction program^54,55^. Contrast transfer function (CTF) estimation was performed using CTFFIND 4.1^56^. All steps of helical reconstruction, three-dimensional (3D) refinement, and post-processing were carried out using RELION 4.0 and RELION 5.0 beta^57,58^. The filaments were picked automatically using Topaz in RELION 4.0^59,60^. Particles were extracted using a box size of 1024 and 300 or 256 pixels with an inter-box distance of 3 asymmetrical units at helical rise of 4.75 Å. 2D classification of 1024-pixel particles was only used to estimate the fibril crossover distance. 2D classifications of 300-pixel particles were used to select suitable particles for further processing. An initial 3D reference model was generated from a subset of 2D class averages using relion_helix_inimodel2d program, as previously described^61^. Fibril helix was assumed to be left-handed for 3D reconstruction. 3D auto-refinement was followed by 3D classification without image alignment to filter out suboptimal classes, after which selected particles underwent further 3D auto-refinement. Subsequent 3D auto refinements with optimization of helical twist and rise were carried out once the estimated resolution of the map reached beyond 4.75 Å. Multiple rounds of CTF refinement, 3D classification (without image alignment), auto-refinement, and post-processing were performed sequentially to reach the highest possible resolution. Throughout reconstruction, C1 symmetry was applied. Although the fibril architecture suggested a possible 2₁ screw symmetry, imposing this symmetry during the final refinement iterations resulted in a deterioration of map quality and reduced resolution. Therefore, C1 symmetry was retained for the final reconstruction to maximize map quality and reliability for structural interpretation. As the optimized rise of all three structures was ~4.91 Å, we performed the final post-processing job in which we calibrated the pixel size to 0.932 Å/px (from 0.954 Å/px), corresponding to the rise of 4.85 Å. Overall resolutions were calculated from Fourier shell correlations at 0.143 between two independently refined half-maps using a soft-edged solvent mask^62^. Local resolutions were estimated using RELION’s Local Resolution tool. Additional processing details are available in Supplementary Table 2.

### Atomic model building and refinement

We used the automated machine-learning ModelAngelo approach with minor modifications to obtain an initial atomic model^63^. First, COOT v0.9.8.1 was used to build the peptide backbone of a single fibril layer featuring all alanine residues^64^. We then created a new density map of one fibril layer in ChimeraX v1.8 using the command ‘vol zone #1 near #2 range 3 new true’, where #1 refers to the post-processed fibril density map and #2 is the peptide backbone model^65^. This new map was then entered ModelAngelo, both with and without the primary ApoA-1 sequence, to obtain the initial atomic models of AApoA-1 fibrils. Using COOT, we made residue modifications and real-space refinements to finalize the model. Further refinement was carried out using ‘phenix.real_space_refine’ from PHENIX 1.20^66^. ChimeraX v1.8 was used for molecular graphics and structural analysis^67^. All refinement statistics are summarized in Supplementary Table 2.

### Stabilization energy calculation

Solvation energy per residue was determined by summing the products of each atom’s buried surface area and its atomic solvation energy^68^. The total chain energy was calculated by summing the residue energies, and each residue was assigned a distinct color in the solvation energy map to visualize variations in energy distribution.

Link to download the program: 10.5281/zenodo.6321286.

### APRs analysis

APRs were predicted using four common prediction models: TANGO^69–71^, WALTZ^72^, Aggrescan^73^, and AmylPred^74^. For each ApoA1 sequence variant (WT, G26R, L90P, and R173P), residues predicted as APRs by a model (“hit”) were given a score of 1, while residues not part of an APR were given a score of 0. This scoring process was repeated across all four models, yielding a per-residue score based on the sum of these hits. For each model, APR criteria were as follows: in TANGO, any 5-residue or longer with a score ≥5.0; in WALTZ, residues with a score >0; in Aggrescan, 5-residue stretches with a score ≥0.02; and in AmylPred, a score of 3 or more. Total scores were recorded in a PDB file as B-factors for visualizing the heatmap model, generated in ChimeraX v1.8^67^.

### Figure panels

Figure panels were created with ChimeraX v1.8 and Adobe Illustrator.

### Reporting summary

Further information on research design is available in the Nature Portfolio Reporting Summary linked to this article.

## Supplementary information

Supplementary Information

Reporting Summary

Transparent Peer Review File

## Source data

Source Data

## Data Availability

Unless otherwise stated, all data supporting the results of this study can be found in the article, supplementary, and source data files. MS data have been deposited to MassIVE database (a member of ProteomeXchange) under accession code MSV000098187. Cryo-EM maps have been deposited in the Electron Microscopy Data Bank under accession codes EMD-71896, EMD-71897 and EMD-71898 for L90P, R173P—heart and R173P—kidney samples, respectively; and the corresponding pdb models are 9PVY (L90P) [https://www.rcsb.org/structure/9PVY], 9PVZ (R173P—heart) [https://www.rcsb.org/structure/9PVZ] and 9PW3 (R173P—kidney) [https://www.rcsb.org/structure/9PW3]. The source data underlying Supplementary Figs. 7 and 8 are provided as a Source Data file. Source data are provided with this paper.
