# Supplementary material for: Cryo-EM reveals structural variability of apolipoprotein A-I amyloid fibrils across organs, mutations, and clinical presentations
Source: Nat Commun. 2026 Apr 17;17:5324. doi: 10.1038/s41467-026-72150-z (PMC13273174; doi:10.1038/s41467-026-72150-z)
Supplement: Supplementary file 1 — Supplementary Information [file 41467_2026_72150_MOESM1_ESM.pdf]

Cryo-EM reveals structural variability of apolipoprotein A-I amyloid fibrils across organs, mutations, and clinical presentations

Authors: Binh An Nguyen[1]*, Maria del Carmen Fernandez-Ramirez[1], Parker Bassett[1], Virender Singh[2], Preeti Singh[1], Maja Pękała[1], Layla Villalon[1], Yasmin Ahmed[1], Andrew Lemoff[3], Bret Evers[4,5], Christian Lopez[6], Barbara Kluve-Beckerman[7], Lorena Saelices[1]*

Affiliations:
[1]Center for Alzheimer's and Neurodegenerative Diseases, Department of Biophysics, Peter O'Donnell Jr Brain Institute, University of Texas Southwestern Medical Center (UTSW), Dallas, TX, USA.
[2]SciKonnect and BioPatriKa, India.
[3]Department of Biochemistry, University of Texas Southwestern Medical Center, Dallas, TX, USA
[4]Department of Pathology, University of Texas Southwestern Medical Center (UTSW), Dallas, TX, USA.
[5]Department of Ophthalmology, University of Texas Southwestern Medical Center (UTSW), Dallas, TX, USA.
[6]Department of Internal Medicine, University of Texas Southwestern Medical Center (UTSW), Dallas, TX, USA.
[7]Department of Pathology and Laboratory Medicine, Indiana University School of Medicine, Indianapolis, IN, USA.

* Correspondence to:
Binh An Nguyen, anbinh.nguyen@utsouthwestern.edu
Lorena Saelices, lorena.saelicesgomez@utsouthwestern.edu

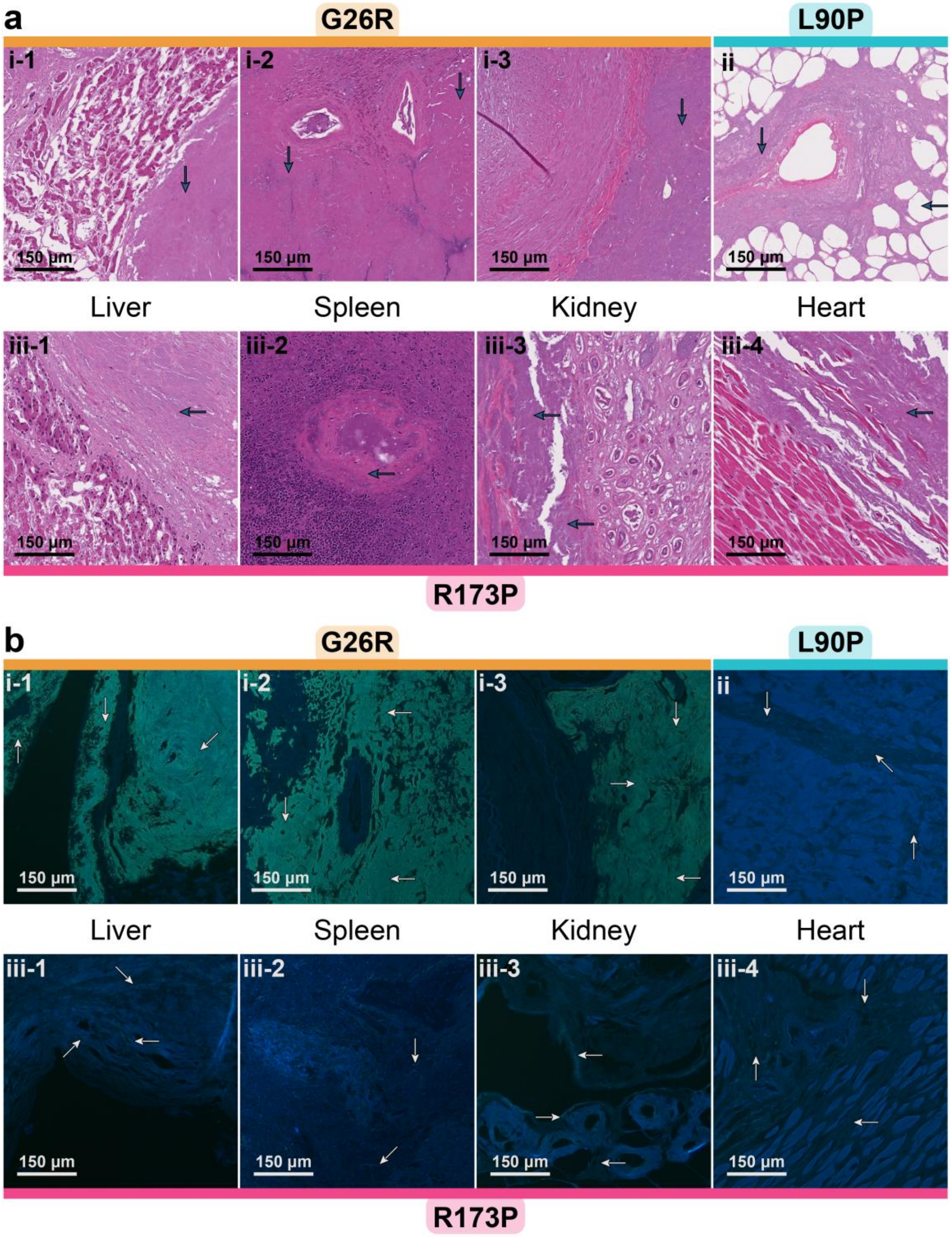

Supplementary Figure 1. Histological analysis of AApoA-I amyloid deposits of various tissue samples from three patients: G26R, L90P and R173P using (**a**) hematoxylin and eosin, and (**b**) Thioflavin S stains. Arrows indicate amyloid deposits. Cross sections show amyloid in the liver, spleen and kidney of G26R (**i1-3**), the heart of L90P (**ii**) and the liver, spleen, kidney and heart of R173P (**iii1-4**). Scale bar: 150 µm.

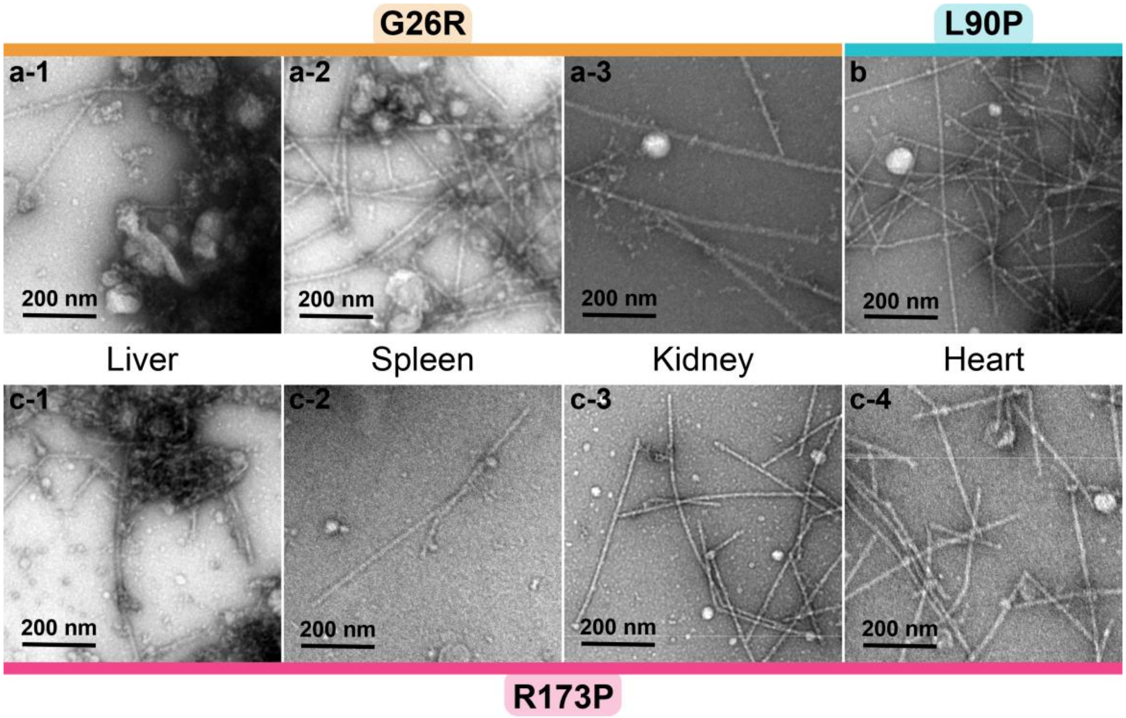

Supplementary Figure 2. Negatively stained transmission electron microscopy (TEM) images of AApoA-I fibrils extracted from various tissue samples in three patients: G26R, L90P and R173P. TEM images of fibrils extracted from the liver, spleen and kidney of G26R (**a1-3**); fibrils from the heart of L90P (**b**); and the liver, spleen, kidney and heart of R173P (**c1-4**). Scale bar: 200 μm.

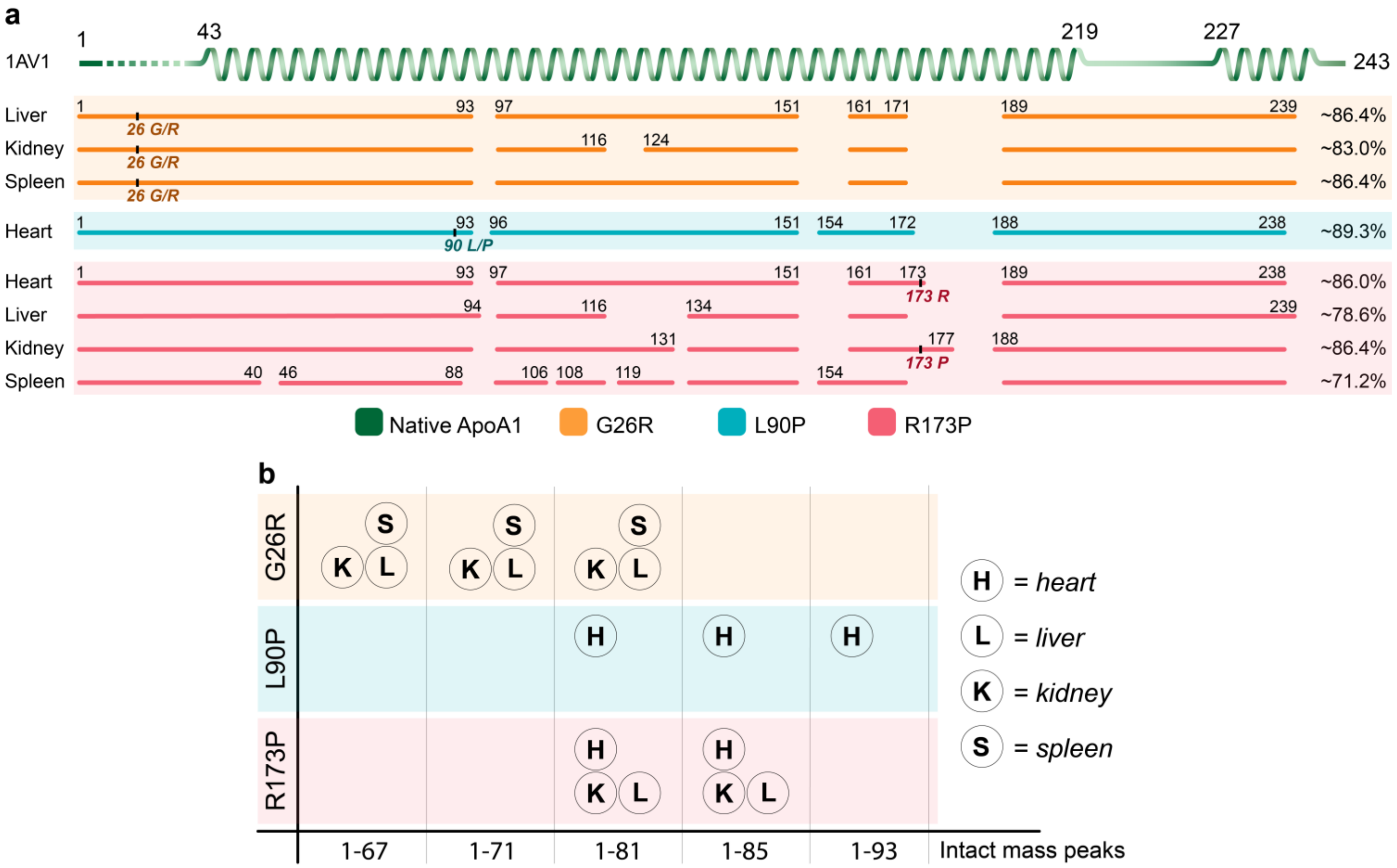

Supplementary Figure 3. Mass spectrometry analysis of fibrils extracted from three AApoA-I patients: G26R, L90P and R173P. (a) Tryptic digest analysis of all fibril samples and GluC analysis for only R173P sample, with the native sequence shown in green (α-helix structure indicated, pdb: 1AV1). Fibril fragments are color-coded by patients: G26R (orange), L90P (blue), and R173P (pink). Detected residues are shown as continuous lines with the corresponding residue positions indicated above each sequence. Variant-specific residues (wild-type and mutant) are noted at their respective positions. Total sequence coverage for each sample is provided at the end. (**b**) Intact mass analysis identifying peaks corresponding to AApoA-I fragments, with fibril samples color-coded as in (a). H, L, K, and S represent fibrils from the heart, liver, kidney, and spleen, respectively.

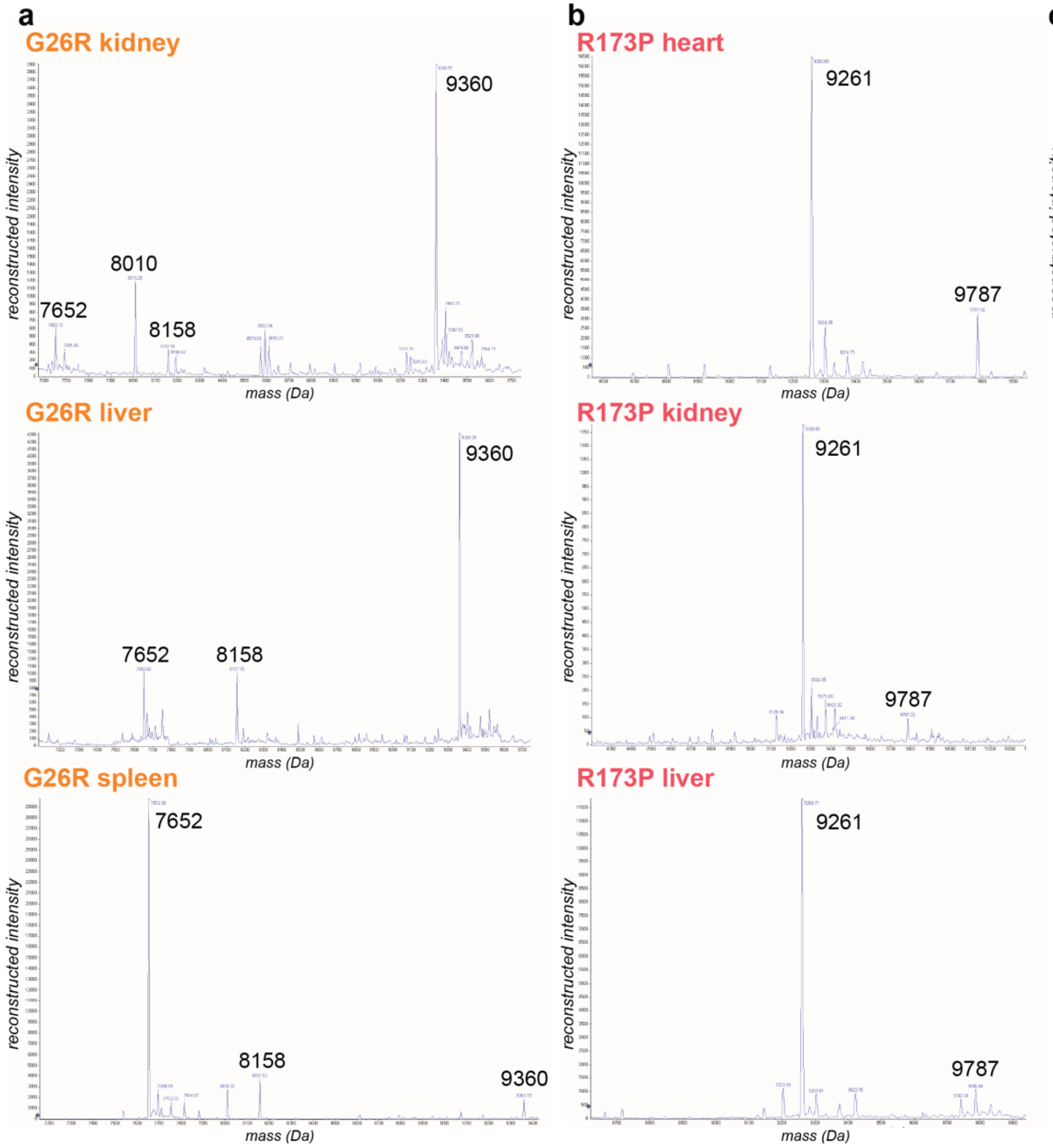

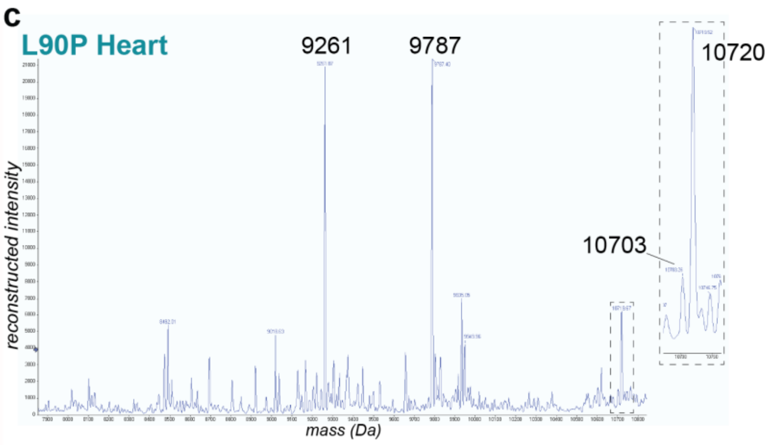

d

peptide fragment	Theoretical mass (Da)	Observed mass (Da) G26R	R173P	L90P
1-93	10720 (WT)			10720
	10703 (L90P)			10703
1-85	9787 (WT)		9787	9787
1-81	9360 (G26R)	9360		
	9261 (WT)		9261	9261
1-71	8158 (G26R)	8158		
	8059 (WT)			
1-67	7652 (G26R)	7652		
	7553 (WT)			

Supplementary Figure 4. Intact mass analysis of AApoA-I fibril samples. (**a-c**) Deconvoluted intact mass spectra showing peaks corresponding to AApoA-I fragments from the extracted fibrils of G26R, R173P and L90P patients, respectively, across different tissues. (**d**) Summary table listing the detected peaks shown in (a-c) and the corresponding AApoA-I fragments. Source data are provided with this paper.

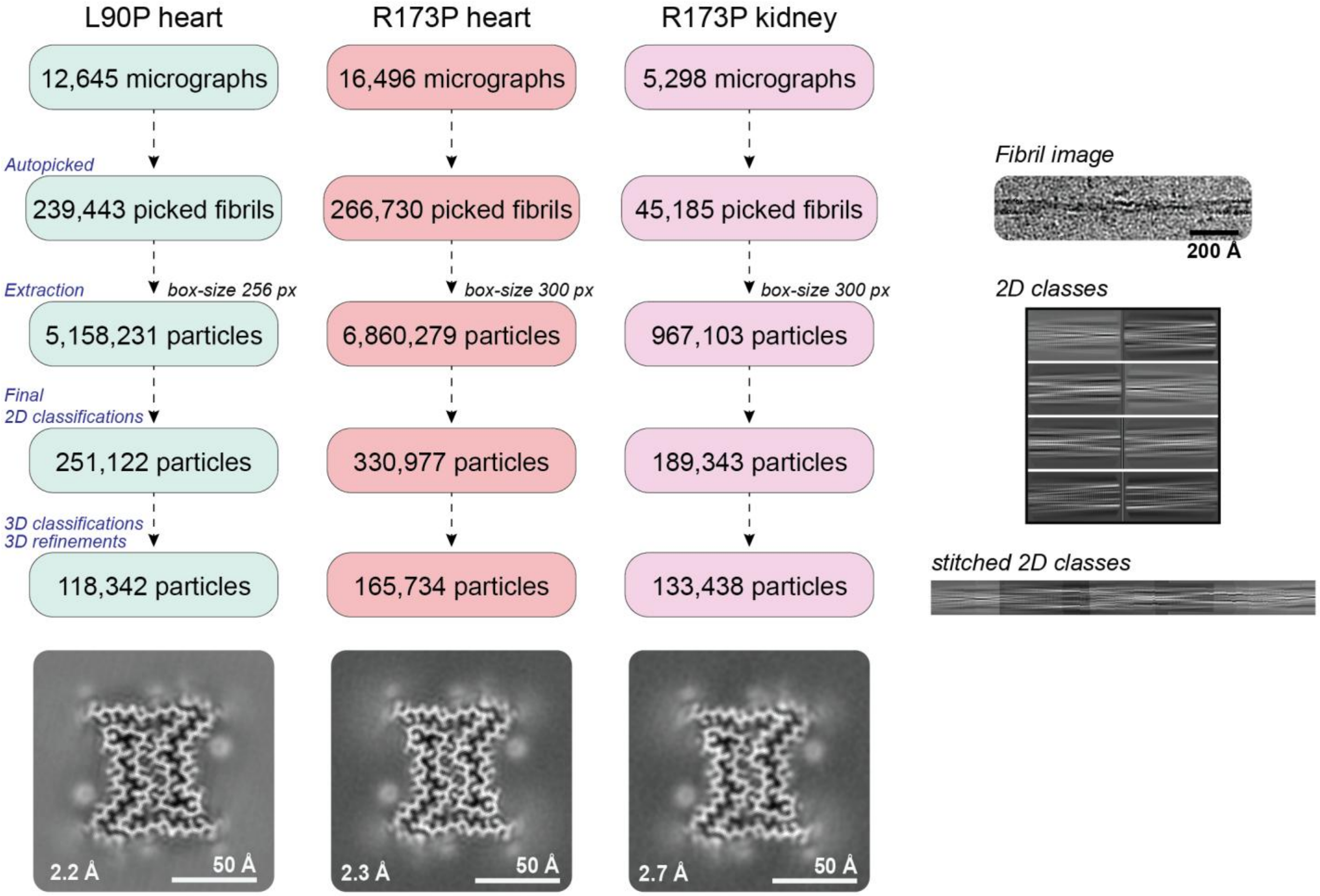

Supplementary Figure 5: Cryo-EM data processing workflow of AApoA-1 fibrils from L90P and R173P samples. A large number of micrographs were collected for three samples, and autopicking were carried out to pick fibrils. Particle extraction with box sizes of 256 px (L90P heart) and 300 px (R173 samples) produced millions of particles which were subjected to 2D classifications. Representative class averages of each particle group are showning, and they are stitched together to determine the crossover which were then used for generating the 3D initial model, 3D classification and Refine3D. Final 3D reconstruction were selected based on the highest resolution and best-defined structural features.

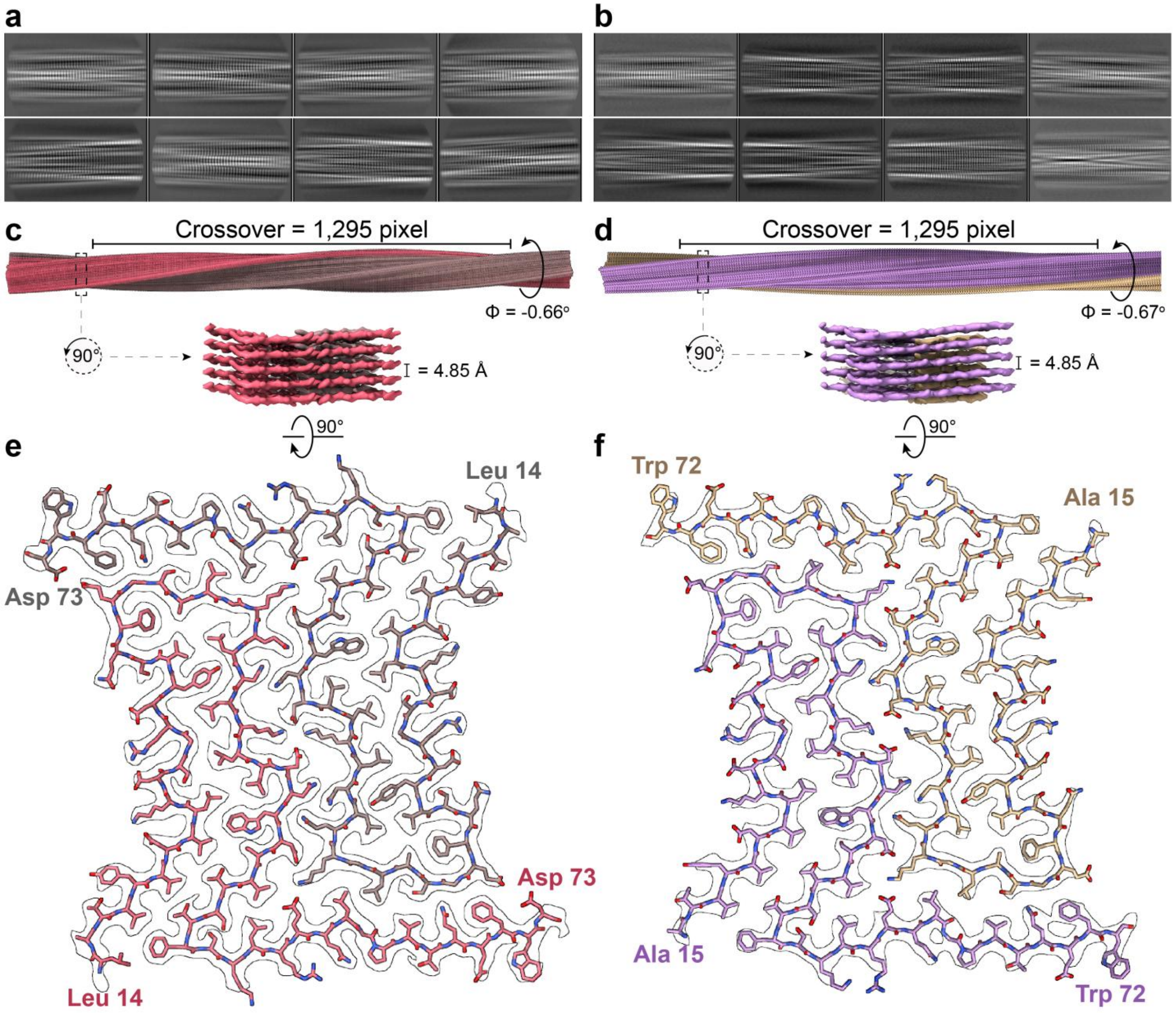

Supplementary Figure 6. Helical reconstructions of AApoA-I fibrils from R173P samples. (a) and (b) 2D class averages of R173P fibrils from the heart and kidney, respectively. (c) and (d) The reconstructed R173P fibrils from the heart and kidney, respectively, containing 360 layers and the magnified view of a subset of 5 fibril layers. (e) and (f) Top view of a single fibril layer from R173P fibrils (heart and kidney, respectively) with a model consisting of two protofilaments spanning residues Leu 14 or Ala 15 to Asp 73 or Trp 72.

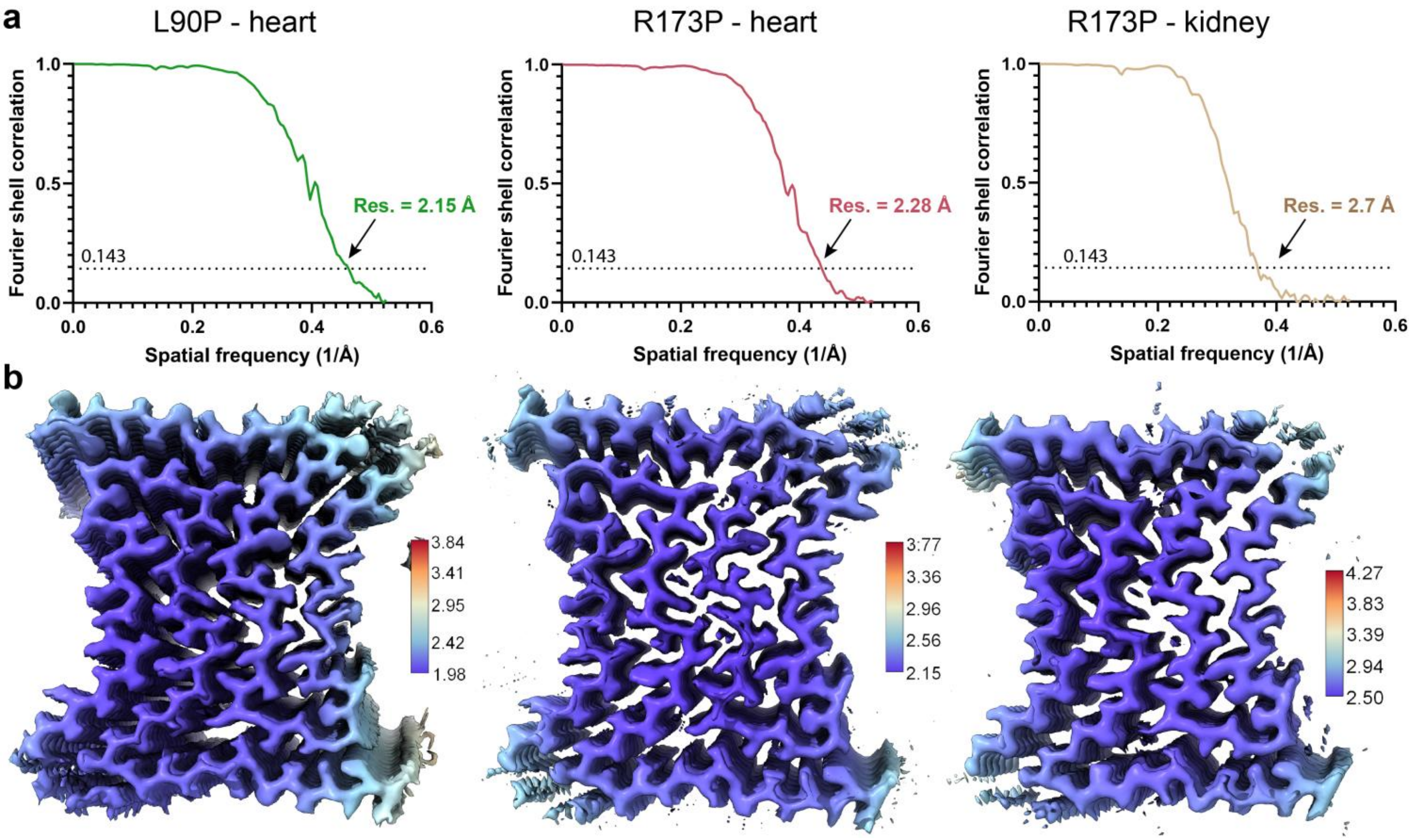

Supplementary Figure 7. FSC curves and local resolution maps of cryo-EM structures from L90P heart, R173P heart and kidney. **a.** Evaluation of the resolution of cryo-EM maps by Fourier shell correlation (FSC) curves of two independently refined half-maps from these samples. **b.** Local resolution estimation for 3D reconstructions of AApoA-I fibrils. Resolution scale in Angstroms. Source data are provided as a Source Data file.

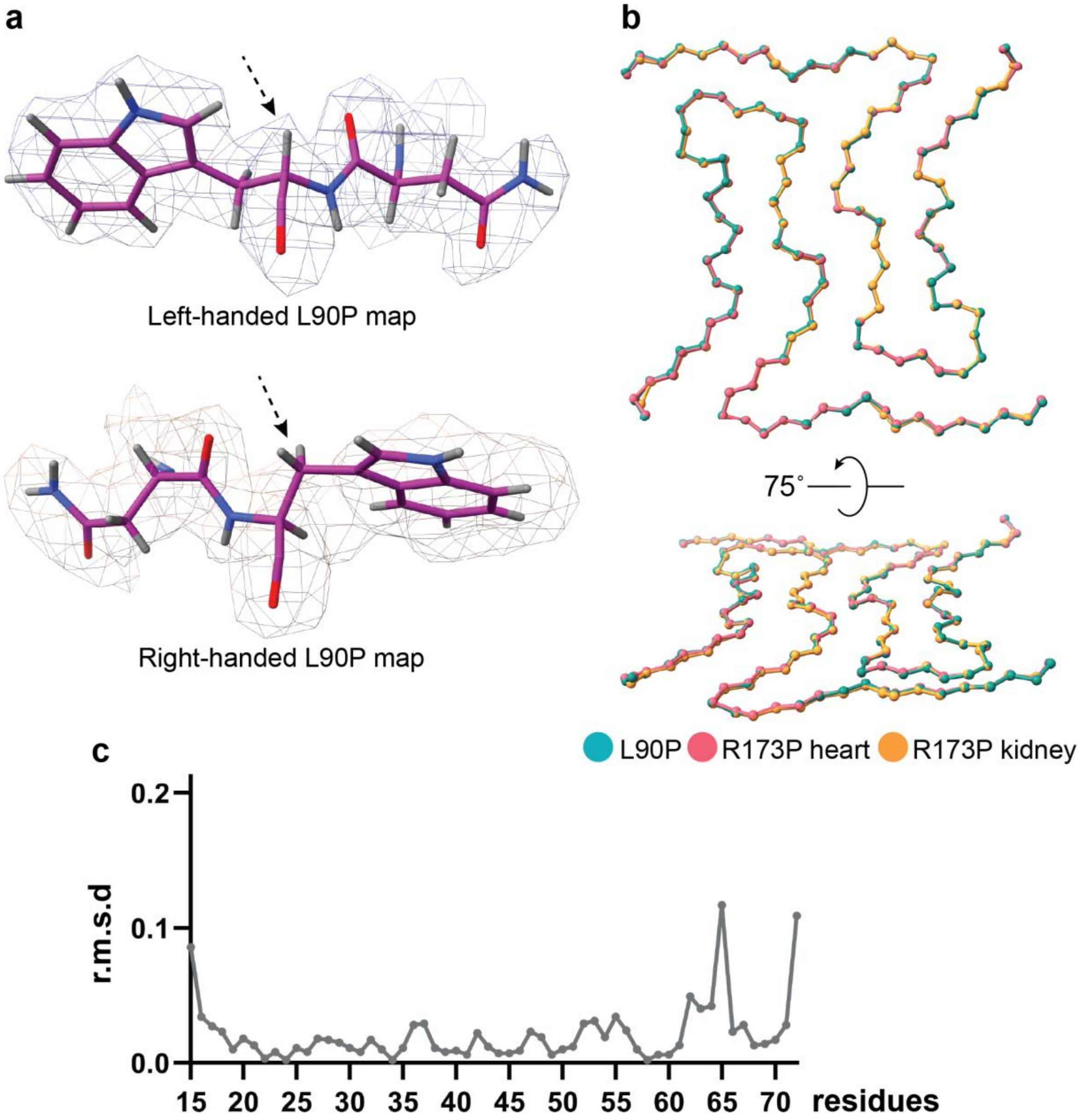

Supplementary Figure 8. Handedness of AApoA-I fibril maps and their structural alignment. (a) representative L-amino acids demonstrate a better fit to the left-handed L90P map compared to the right-handed map. **(b)** Top view and tilted view of structural alignment of the Cα backbones from L90P fibrils (teal) and R173P fibrils from heart (light red) and kidney (yellow) samples. **(c)** Comparison of Cα displacement among these three structures, estimated using GESAMT, shows that the majority of displacements are less than 0.1 Å compared to the consensus sequence of the three fibril structures, revealing highly structural similarity. Source data are provided as a Source Data file.

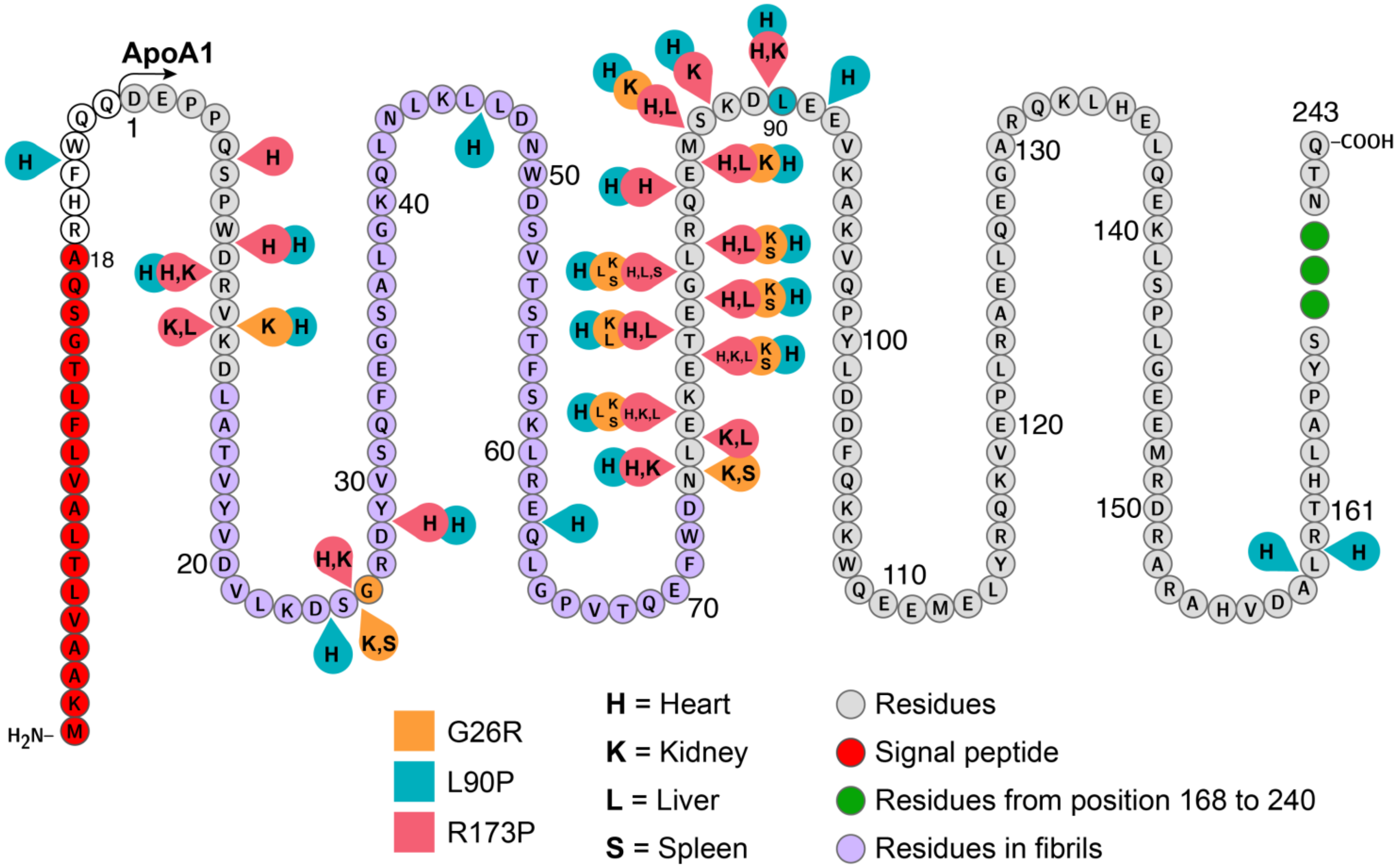

Supplementary Figure 9. Combined proteolytic sites from all three variants analyzed by mass-spectrometry. The signal peptide is highlighted in red. Regions corresponding to G26R, L90P and R173P variants are shown in light orange, blue, and salmon pink, respectively. The fibril core is depicted in purple, and residues from positions 168 to 240 are shown in green. Proteolytic sites identified in the heart, kidney, liver and spleen are denoted by H, K, L, and S, respectively.

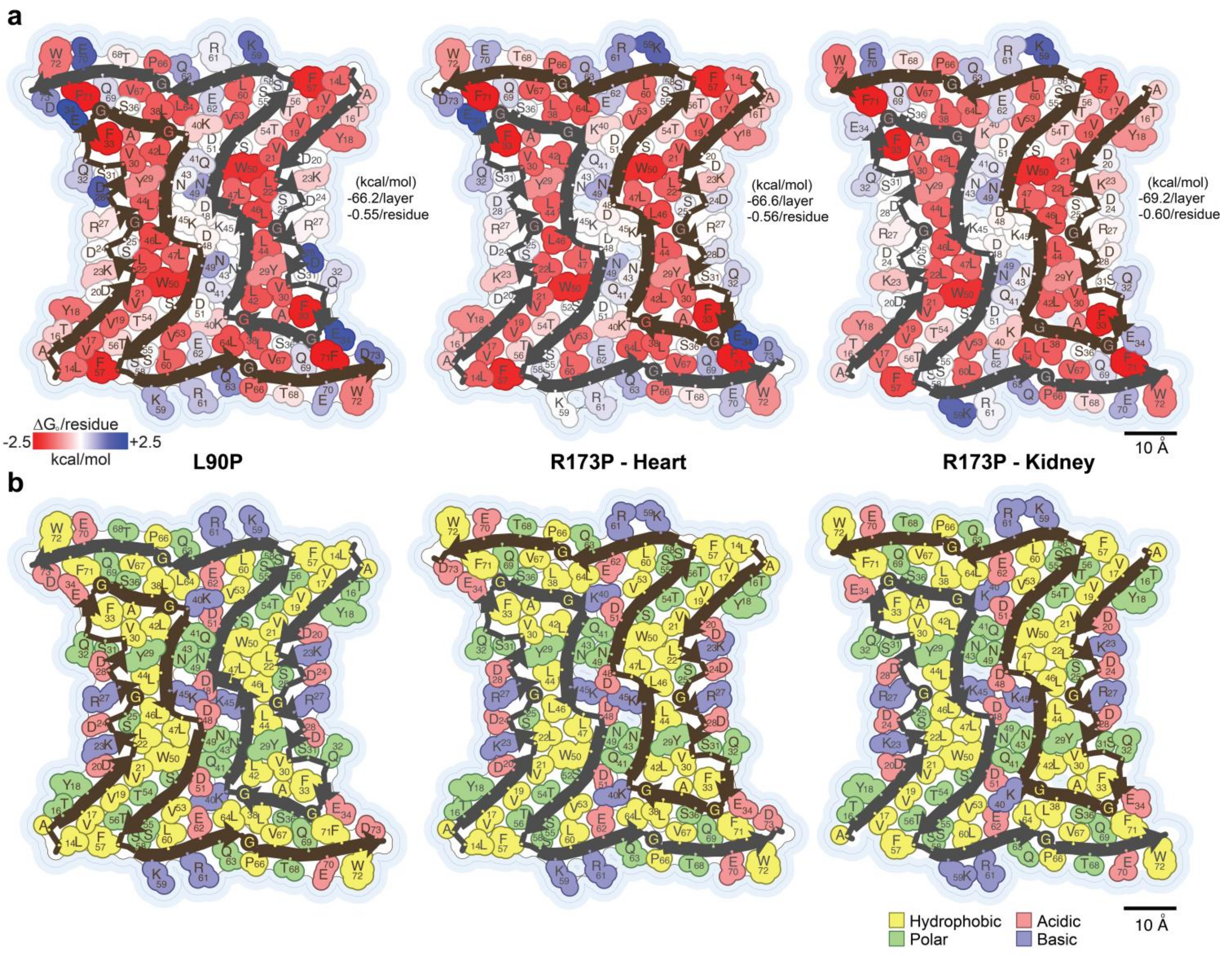

Supplementary Figure 10. **Solvation energy and fibril composition profile of L90P and R173P amyloidosis**. **(a)** Residue solvation energies profiles for fibril structures from the L90P heart, R173P heart, and R173P kidney, with stabilization energies ranging from favorable (red, -2.5 kcal/mol) to unfavorable (blue, 2.5 kcal/mol). Scale: 10 Å. **(b)** Residue composition profiles of fibril structures from the same samples, with residue color-coded as hydrophobic (yellow), polar (green), acidic (red), and basic (blue). Scale, 10 Å.

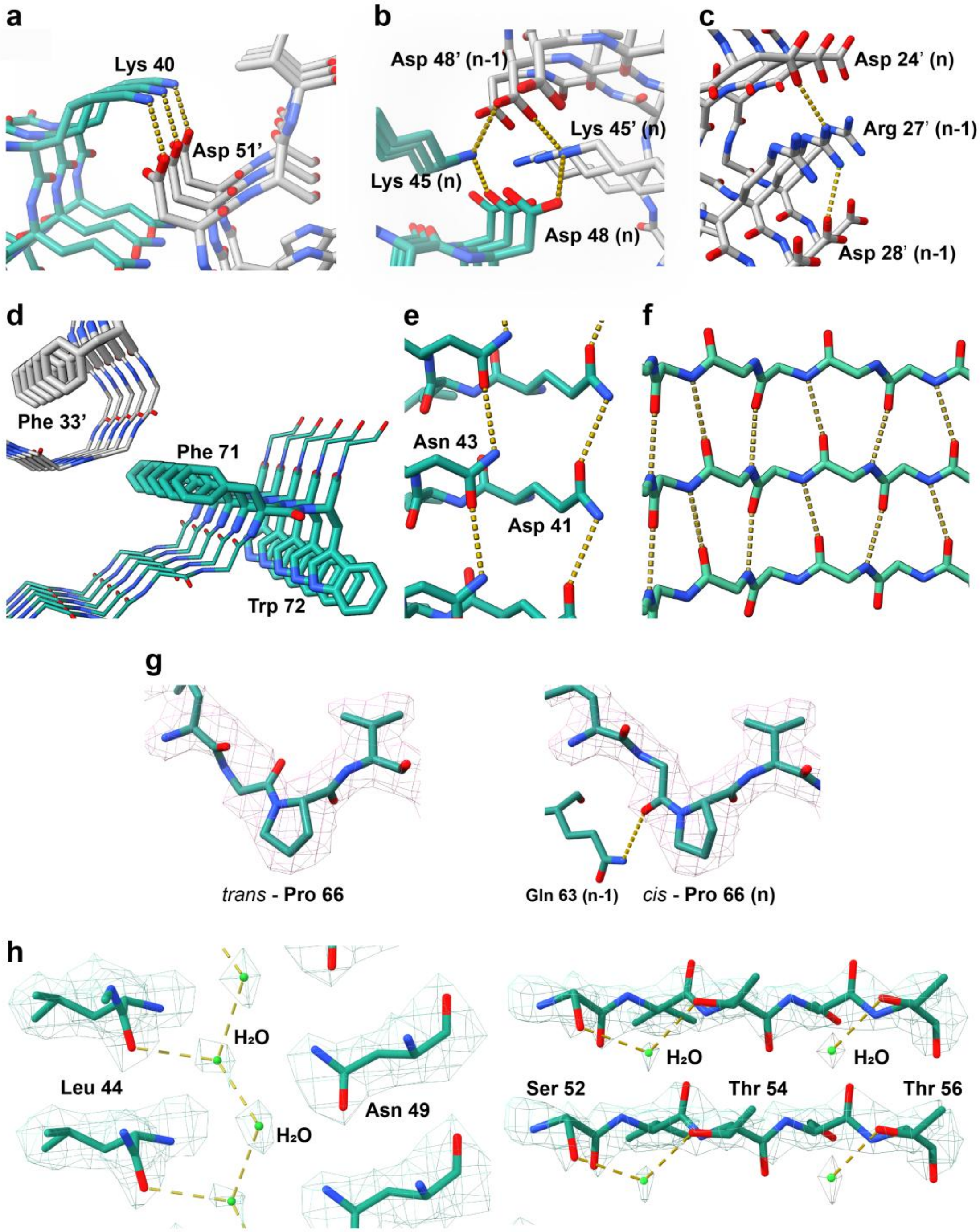

Supplementary Figure 11. Molecular interactions stabilize AApoA-I fibril structure, the cis-isomer Pro 66 and densities of likely water molecules. (**a**) Salt bridges (approximately 2.7 Å) formed between Lys 40 on layer n and Asp 51’ on layer n-1 of the adjacent protofilament. (**b**) Salt bridges (approximately 2.5 Å) formed between Lys 45 on layer n and Asp 48’ and Asp 48 on layer n-1. (**c**) Salt bridges formed within the same layer (n-1) between Arg 27’ and Asp 28, and between Arg 27’ of layer n-1 and Asp 24’ of layer n. (**d**) π-π interactions from stacking side chains of aromatic residues. (**e, f**) H-bonds formed by layer-stacking aspartate and glutamate side chains, and peptide backbone, respectively. (**g**) Density map and the model of trans-Pro 66 (on the left) and cis-Pro 66 (on the right) with the additional hydrogen bond with Gln 63 (n-1). (**h**) Inter-layer water molecules forming hydrogen bonds with each other and with Leu 44 along the fibril axis (on the left) and intra-layer water molecules forming hydrogen bonds with Ser 52 and Thr 54 (on the right).

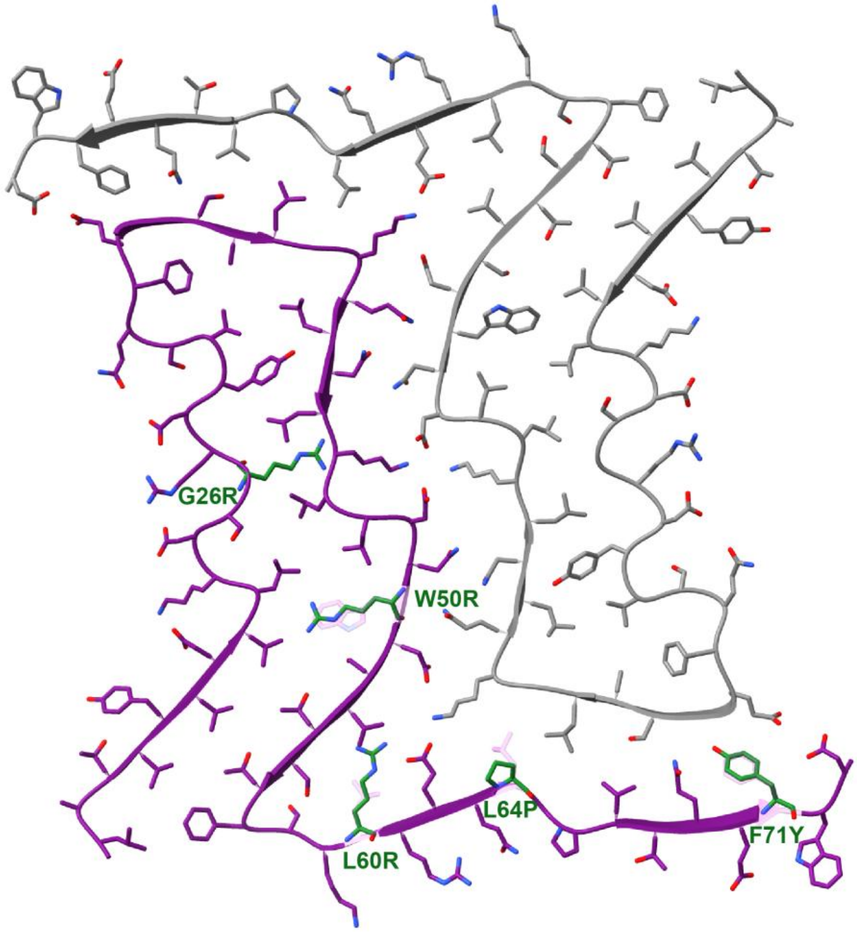

Supplement Figure 12. Schematic representation illustrates how the introduction of N-terminal mutations into the fibrils core of L90P and R173P samples may influence fibril structures. These mutations could potentially alter the structural integrity and stability of the fibrils due to changes of side chain properties such as bulkiness, charge, or rigidity. Mutated residues are colored green and native residues are dimmed in the background.

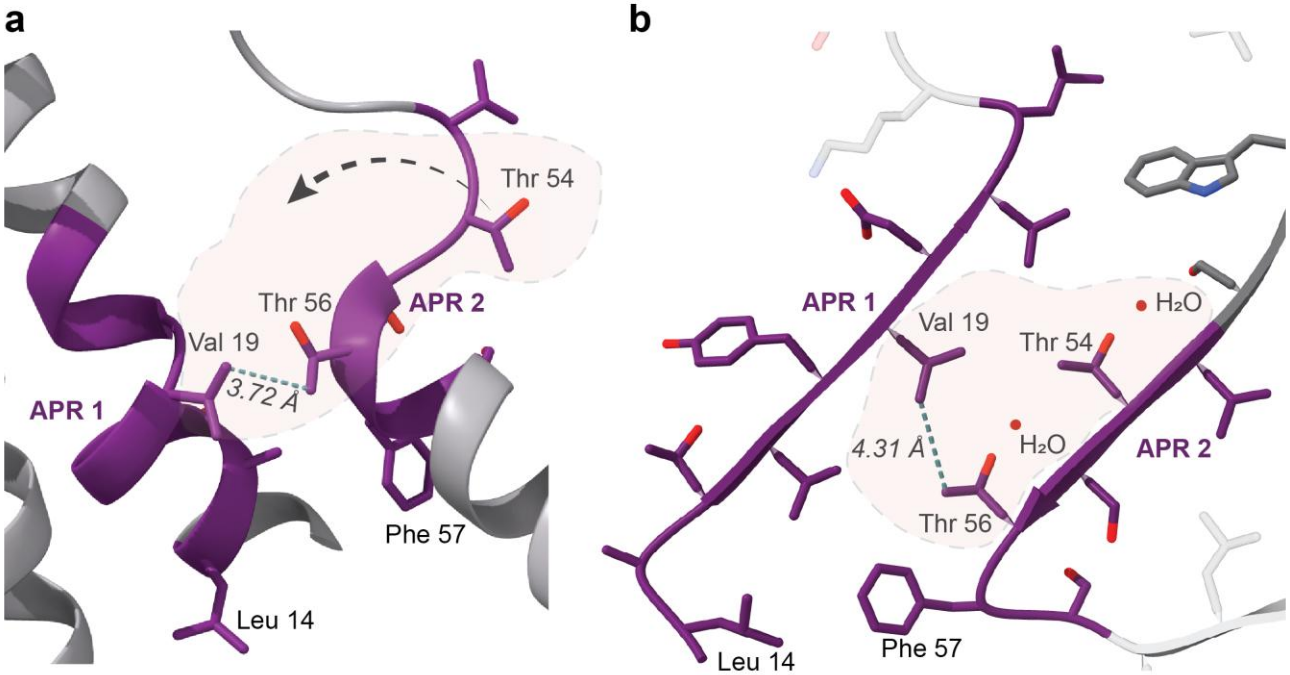

Supplementary Figure 13: The two APR (1 and 2, in purple) of ApoA-I are spatially adjacent in both the native and fibril structure of the L90P, R173P samples. **(a)** In the native crystal structure (PDB: 3R2P), APR1 and APR2 are positioned near each other, with Val 19 on APR1 located 3.72 Å from Thr 56 on APR2. **(b)** In the fibril structures, APR1 and APR2 remain close, with Val 19 on APR1 being 4.31 Å from Thr 56 on APR2.

Supplement Table 1: ApoA-I variants and the corresponding clinical manifestations [1-4].

Variants	Amyloid in kidney biopsy	Major organ involvement
D20Y	No	Esophageal
G26R	Yes	Renal failure
E34K	Yes	Renal failure
W50R	Yes	Renal failure
L60R	Yes	Renal failure
∆60-71/60V61T	Yes	Hepatic failure
L64P	Yes	Renal failure
∆70-72	Yes	Renal failure
F71Y	n/r	Hepatic involvement
N74fs	n/r	Renal involvement
L75P	Yes	Renal failure
L90P	n/r	Cardiac failure
∆K107	n/r	Cardiomyopathy
A154fs	Yes	n/r
H155Mfs	Yes	Renal failure
L170P	n/r	Laryngeal
Q172P	n/r	Cardiac failure
R173P	n/r	Cardiomyopathy
L174S	n/r	Cardiac failure
A175P	n/r	Laryngeal
L178(H/R)	n/r	Cardiomyopathy

n/r = not report. Purple represents the fibril core. Light blue represents the C-terminal outside of fibril core.

Supplement Table 2: List of tryptic and semi-tryptic ApoA-I peptides [P02647] from MS analysis

Annotated Sequence	#PSMs	Positions in Protein	G26R kidney	G26R liver	G26R spleen
[-Q].DEPPQSPWDRVK.[D]	15	P02647 [25-36]	7.93E+06	8.25E+05	4.24E+06
[R].VKDLATVYVDVLK.[D]	150	P02647 [35-47]	1.53E+09	4.02E+08	---
[R].VKDLATVYYVDVLKDSR*[R]	10	P02647 [35-50]	---	---	1.36E+07
[R].VKDLATVYYVDVLKDSG*R.[D]	1	P02647 [35-51]	---	6.91E+05	---
[R].VKDLATVYYVDVLKDSR*R.[D]	1	P02647 [35-51]	---	5.52E+05	---
[K].DLATVYYVDVLKDS**R***R.[D]	7	P02647 [37-51]	3.48E+06	---	---
[S].**G***RDYVSQFEGSALGK.[Q]	5	P02647 [50-64]	6.50E+06	3.01E+08	4.75E+06
[R].DYVSQFEGSALGKQLNLK.[L]	2	P02647 [52-69]	1.40E+06	---	5.97E+05
[Q].FEGSALGKQLNLKLLDNWDSVTSTFSK.[L]	1	P02647 [57-83]	---	2.11E+05	---
[K].QLNLKLLDNWDSVTSTFSK.[L]	3	P02647 [65-83]	---	---	1.55E+06
[K].LLDNWDSVTSTFSKLR.[E]	4	P02647 [70-85]	3.21E+06	1.29E+06	1.29E+09
[K].LREQLGPVTQEFWDNLEKETEG.[L]	17	P02647 [84-105]	2.02E+08	6.59E+07	3.47E+07
[K].LREQLGPVTQEFWDNLEKETEGLR.[Q]	8	P02647 [84-107]	4.22E+06	1.33E+06	---
[K].LREQLGPVTQEFWDNLEKETEGLRQE.[M]	3	P02647 [84-109]	1.70E+06	---	---
[K].LREQLGPVTQEFWDNLEKETEGLRQEM.[S]	2	P02647 [84-110]	5.29E+05	---	---
[K].LREQLGPVTQEFWDNLEKETEGLRQEMSK.[D]	2	P02647 [84-112]	---	6.59E+07	---
[R].EQLGPVTQEFWDNLEKETEG.[L]	15	P02647 [86-105]	4.69E+07	2.71E+07	---
[R].EQLGPVTQEFWDNLEKETEGLRQEM.[S]	2	P02647 [86-110]	8.63E+05	---	---
[R].EQLGPVTQEFWDNLEKETEGLRQEMSK.[D]	1	P02647 [86-112]	---	---	6.24E+05
[K].ETEGLRQEMSKDLEE.[V]	8	P02647 [102-116]	4.30E+06	---	---
[Q].EMSKDLEEVK.[A]	1	P02647 [109-118]	---	3.01E+06	5.79E+05
[K].VQPYLDDFQK.[K]	3	P02647 [121-130]	4.21E+06	2.75E+07	1.12E+07
[K].VQPYLDDFQKK.[W]	1	P02647 [121-131]	6.45E+05	1.98E+07	1.67E+06
[K].KWQEEMELYR.[Q]	1	P02647 [131-140]	4.76E+05	2.33E+06	2.46E+06
[K].WQEEMELYR.[Q]	2	P02647 [132-140]	3.64E+06	1.50E+07	---
[R].QKVEPLRAELQEGAR.[Q]	1	P02647 [141-155]	---	5.59E+05	9.41E+05
[R].AELQEGAR.[Q]	1	P02647 [148-155]	4.93E+06	2.71E+07	9.73E+06
[R].QKLHELQEK.[L]	1	P02647 [156-164]	---	3.53E+06	8.06E+05
[K].LSPLGEEMRDR.[A]	1	P02647 [165-175]	7.37E+06	5.54E+07	7.46E+06
[R].THLAPYSDELR.[Q]	7	P02647 [185-195]	3.51E+07	4.49E+07	3.18E+07
R].LEALKENGGAR.[L]	1	P02647 [185-197]	---	---	2.37E+05
[R].LAEYHAK.[A]	1	P02647 [202-212]	---	---	---
[R].LAEYHAK.[A]	2	P02647 [213-219]	2.28E+07	2.51E+07	7.02E+06
[K].ATEHLSTLSEK.[A]	5	P02647 [220-230]	6.12E+07	7.27E+07	3.28E+07
[K].AKPALEDLR.[Q]	1	P02647 [231-239]	2.48E+06	6.93E+06	4.31E+06
[R].QGLLPVLESFK.[V]	2	P02647 [240-250]	8.25E+06	9.72E+06	4.89E+06
[K].VSFLSALEEYTK.[K]	4	P02647 [251-262]	3.17E+07	2.55E+07	1.81E+07
[K].VSFLSALEEYTKK.[L]	1	P02647 [251-263]	4.36E+05	5.40E+05	4.75E+05

Annotated Sequence	#PSMs	Positions in Protein	L90P heart		
[-Q].DEPPQSPWDR.[V]	104	P02647 [25-34]	2.42E+09		
[R].VKDLATVYVDVLK.[D]	305	P02647 [35-47]	9.66E+09		
[R].VKDLATVYYVDVLKDSGRDYVSQFEGSALGK.[Q]	7	P02647 [35-64]	2.04E+07		
[R].DYVSQFEGSALGKQLNLK.[L]	4	P02647 [52-69]	1.83E+07		
[K].LLDNWDSVTSTFSK.[L]	156	P02647 [70-83]	7.34E+09		
[K].LREQLGPVTQEFWDNLEKETEGLRQEMSK.[D]	10	P02647 [84-112]	2.18E+07		
[R].EQLGPVTQEFWDNLEKETEGLRQEMSKD**L***E.[E]	2	P02647 [86-115]	1.00E+06		
[R].QEMSKDP*EEV.[K]	3	P02647 [108-117]	1.20E+08		
[A].KVQPYLDDFQK.[K]	3	P02647 [120-130]	6.56E+06		
[K].VQPYLDDFQK.[K]	9	P02647 [121-130]	1.57E+08		
[K].KWQEEMELYR.[Q]	4	P02647 [131-140]	2.64E+07		
[R].QKVEPLRAELQEGAR.[Q]	4	P02647 [141-155]	2.50E+07		
[R].QKLHELQEK.[L]	4	P02647 [156-164]	3.01E+07		
[K].LSPLGEEMRDR.[A]	5	P02647 [165-175]	3.34E+08		
[R].AHVDALRTHLAPYSDELR.[Q]	4	P02647 [178-195]	2.94E+06		
[A].RLAEYHAK.[A]	1	P02647 [212-219]	5.74E+06		
[R].LAEYHAK.[A]	4	P02647 [213-219]	5.18E+08		
[K].ATEHLSTLSEK.[A]	18	P02647 [220-230]	2.25E+09		
[K].AKPALEDLRQGLLPVLESFK.[V]	2	P02647 [231-250]	1.21E+06		
[K].VSFLSALEEYTK.[K]	18	P02647 [251-262]	1.34E+09		
[K].VSFLSALEEYTKK.[L]	3	P02647 [251-263]	9.35E+06		

Annotated Sequence	#PSMs	Positions in Protein	R173P heart	R173P kidney	R173P liver
[Q].DEPPQSPWDR.[V]	55	P02647 [25-34]	1.38E+07	1.95E+08	1.19E+08
[D].RVKDLATVYVDVLK.[D]	12	P02647 [34-47]	5.45E+06	4.32E+06	6.77E+08
[R].VKDLATVYVDVLK.[D]	97	P02647 [35-47]	9.44E+08	2.85E+09	---
[K].DSGRDYVSQFEGSALGK.[Q]	42	P02647 [48-64]	8.17E+07	1.14E+09	3.64E+08
[K].QLNLKLLDNWDSVTSTFSK.[L]	2	P02647 [65-83]	3.95E+06	1.19E+06	5.11E+05
[K].LLDNWDSVTSTFSK.[L]	62	P02647 [70-83]	2.06E+08	3.50E+09	1.07E+09
[K].LREQLGPVTQEFWDNLEKETEGLRQE.[M]	23	P02647 [84-109]	4.76E+07	3.77E+07	8.67E+06
[K].ETEGLRQEMSKDLEEV.[K]	5	P02647 [102-117]	1.81E+06	3.06E+06	2.17E+06
[R].QEMSKDLEEVK.[A]	1	P02647 [108-118]	---	---	2.75E+05
[K].VQPYLDDFQK.[K]	7	P02647 [121-130]	3.78E+07	9.61E+06	---
[K].VQPYLDDFQKK.[W]	3	P02647 [121-131]	8.29E+06	6.05E+05	1.25E+06
[K].KWQEEMELYR.[Q]	4	P02647 [131-140]	1.02E+07	7.13E+05	2.92E+06
[R].QKVEPLRAELQEGAR.[Q]	2	P02647 [141-155]	1.00E+06	2.30E+05	---
[K].VEPLRAELQEGAR.[Q]	5	P02647 [143-155]	3.60E+06	1.17E+06	---
[K].LHELQEK.[L]			---	2.56E+06	5.07E+06
[R].QKLHELQEK.[L]	1	P02647 [156-164]	---	---	---
[K].LSPLGEEMRDR.[A]	4	P02647 [165-175]	2.94E+07	7.55E+06	7.65E+06
[R].THLAPYSDELR.[Q]	4	P02647 [185-195]	1.27E+07	5.05E+06	2.18E+06
[R].THLAPYSDELRQP*LAAR.[L]	2	P02647 [185-201]	1.59E+06	---	1.59E+06
[R].LAEYHAK.[A]	4	P02647 [213-219]	7.68E+06	2.69E+05	8.66E+06
[K].ATEHLSTLSEK.[A]	2	P02647 [220-230]	6.97E+06	1.83E+07	2.95E+07
[K].AKPALEDLR.[Q]	3	P02647 [231-239]	1.62E+06	5.00E+07	3.51E+06
[R].QGLLPVLESFK.[V]	5	P02647 [240-250]	1.57E+07	5.33E+06	6.52E+06
[K].VSFLSALEEYTK.[K]	1	P02647 [251-262]	7.24E+07	7.61E+06	1.98E+07
[K].VSFLSALEEYTKK.[L]		P02647 [251-263]	3.34E+05	1.21E+08	4.44E+05

Supplementary Data Table 3: Cryo-EM data collection, refinement and validation statistics

	AApoA-I-G26R kidney	AApoA-I-G26R spleen	AApoA-I-L90P heart (EMDB-71896) (PDB 9PVY)	AApoA-I-R173P heart (EMDB-71897) (PDB 9PVZ)	AApoA-I-R173P kidney (EMDB-71898) (PDB 9PW3)
**Data collection and processing**					
Microscope	Titan Krios G3i	Titan Krios G3i	Titan Krios G3i	Titan Krios G3i	Titan Krios G3i
Detector	Falcon 4i	Falcon 4i	Falcon 4i	Falcon 4i	Falcon 4i
Magnification	130,000	130,000	130,000	130,000	130,000
Voltage (kV)	300	300	300	300	300
Exposure time (s)	4.05	4.20	4.16	4.19	3.54
Electron exposure (e-/Å^2)	40	40	40	40	40
Defocus range (μm)	-0.8 to -2.6	-0.8 to -2.6	-0.8 to -2.6	-0.8 to -2.6	-0.8 to -2.6
Pixel size (Å/px) (Original/Calibrated)	0.954/0.932	0.954/0.932	0.954/0.932	0.954/0.932	0.954/0.932
**Reconstruction**					
Micrographs	16,361	13,044	12,645	16,496	5,298
Box size (pixel)	1,024	1,024	256	300	300
Initial segments (no.)	1,334,546	632,144	5,158,231	6,860,279	967,103
Final segments (no.)	n/a	n/a	118,342	165,734	133,438
Symmetry imposed	n/a	n/a	C1	C1	C1
Helical rise (Å)	n/a	n/a	4.85	4.85	4.85
Helical twist (°)	n/a	n/a	-0.68	-0.66	-0.67
Map resolution (Å) (FSC = 0.143)	n/a	n/a	2.15	2.29	2.73
Map sharpening *B* factor (Å^2)	n/a	n/a	-66.47	-77.32	-96.14
**Model**					
Initial model used (PDB code)	n/a	n/a	*De novo*	*De novo*	*De novo*
Model resolution (Å) (FSC = 0.143)	n/a	n/a	2.15	2.29	2.72
**Model composition**					
Chains	n/a	n/a	10	10	10
Non-hydrogen atoms	n/a	n/a	4810	4,810	4,650
Protein residues	n/a	n/a	600	600	580
**R.M.S. deviations**					
Bond lengths (Å)	n/a	n/a	0.003	0.003	0.003
Bond angles (°)	n/a	n/a	0.767	0.691	0.668
**Validation**					
MolProbity score	n/a	n/a	1.99	1.86	2.02
Clashscore	n/a	n/a	11.70	7.59	24.95
Poor rotamers (%)	n/a	n/a	0	0	0
**Ramachandran plot**					
Favored (%)	n/a	n/a	93.97	93.10	97.32
Allowed (%)	n/a	n/a	6.03	6.90	2.68
Disallowed (%)	n/a	n/a	0	0	0

Reference

1. Del Giudice, R., Lindvall, M., Nilsson, O., Monti, D.M. & Lagerstedt, J.O. The Apparent Organ-Specificity of Amyloidogenic ApoA-I Variants Is Linked to Tissue-Specific Extracellular Matrix Components. *Int J Mol Sci* **24**(2022).
2. Lu, C., *et al.* Apolipoprotein A-1-related amyloidosis 2 case reports and review of the literature. *Medicine (Baltimore)* **96**, e8148 (2017).
3. Mucchiano, G.I., Häggqvist, B., Sletten, K. & Westermark, P. Apolipoprotein A-1-derived amyloid in atherosclerotic plaques of the human aorta. *J Pathol* **193**, 270-275 (2001).
4. Prokaeva, T., *et al.* An unusual phenotype of hereditary AApoAI amyloidosis caused by a novel Asp20Tyr substitution is linked to pH-dependent aggregation of apolipoprotein A-I. *Biochimica et Biophysica Acta (BBA) - Molecular Basis of Disease* **1871**, 167820 (2025).
