# Supplementary material for: Cryo-EM reveals structural variability of apolipoprotein A-I amyloid fibrils across organs, mutations, and clinical presentations
Source: Nat Commun. 2026 Apr 17;17:5324. doi: 10.1038/s41467-026-72150-z (PMC13273174; doi:10.1038/s41467-026-72150-z)
Supplement: Supplementary file 2 — Reporting Summary [file 41467_2026_72150_MOESM2_ESM.pdf]

nature portfolio

Corresponding author(s): Lorena Saelices

Last updated by author(s): Jan 15, 2026

Reporting Summary

Nature Portfolio wishes to improve the reproducibility of the work that we publish. This form provides structure for consistency and transparency in reporting. For further information on Nature Portfolio policies, see our Editorial Policies and the Editorial Policy Checklist.

Statistics

For all statistical analyses, confirm that the following items are present in the figure legend, table legend, main text, or Methods section.

n/a	Confirmed	
☐	☒	The exact sample size (*n*) for each experimental group/condition, given as a discrete number and unit of measurement
☒	☐	A statement on whether measurements were taken from distinct samples or whether the same sample was measured repeatedly
☒	☐	The statistical test(s) used AND whether they are one- or two-sided<br>*Only common tests should be described solely by name; describe more complex techniques in the Methods section.*
☒	☐	A description of all covariates tested
☒	☐	A description of any assumptions or corrections, such as tests of normality and adjustment for multiple comparisons
☐	☒	A full description of the statistical parameters including central tendency (e.g. means) or other basic estimates (e.g. regression coefficient) AND variation (e.g. standard deviation) or associated estimates of uncertainty (e.g. confidence intervals)
☒	☐	For null hypothesis testing, the test statistic (e.g. *F*, *t*, *r*) with confidence intervals, effect sizes, degrees of freedom and *P* value noted<br>*Give P values as exact values whenever suitable.*
☒	☐	For Bayesian analysis, information on the choice of priors and Markov chain Monte Carlo settings
☒	☐	For hierarchical and complex designs, identification of the appropriate level for tests and full reporting of outcomes
☒	☐	Estimates of effect sizes (e.g. Cohen's *d*, Pearson's *r*), indicating how they were calculated

Our web collection on statistics for biologists contains articles on many of the points above.

Software and code

Policy information about availability of computer code

Data collection	EPU and SerialEM
Data analysis	Relion 4.0 (MRC, LMB Cambridge), CTFFIND4.1 (Rohou, A. & Grigorieff), COOT (Emsley et al., 2010) and PHENIX (Afonine et al., 2018 for Cryo-EM and graphPad prism 9.0 for rest of the analysis, Adobe illustrator for diagrams.

For manuscripts utilizing custom algorithms or software that are central to the research but not yet described in published literature, software must be made available to editors and reviewers. We strongly encourage code deposition in a community repository (e.g. GitHub). See the Nature Portfolio guidelines for submitting code & software for further information.

Data

Policy information about availability of data

All manuscripts must include a data availability statement. This statement should provide the following information, where applicable:

- Accession codes, unique identifiers, or web links for publicly available datasets
- A description of any restrictions on data availability
- For clinical datasets or third party data, please ensure that the statement adheres to our policy

MS data have been deposited to MassIVE database (a member of ProteomeXchange) under accession code MSV000098187 [ftp://massive-ftp.ucsd.edu/v10/MSV000098187/]. Cryo-EM maps have been deposited in the Electron Microscopy Data Bank under accession codes EMD-71896 [https://www.ebi.ac.uk/emdb/EMD-71896], EMD-71897 [https://www.ebi.ac.uk/emdb/EMD-71897] and EMD-71898 https://www.ebi.ac.uk/emdb/EMD-71898] for L90P, R173P – heart and R173P – kidney samples, respectively; and the corresponding pdb models are 9PVY (L90P) [https://www.rcsb.org/structure/9PVY], 9PVZ (R173P – heart) [https://

www.rcsb.org/structure/9PVZ] and 9PW3 (R173P – kidney) [https://www.rcsb.org/structure/9PW3]. The source data underlying Supplementary Figures 6 and 7 are provided as a Source Data file. Source data are provided with this paper.

Research involving human participants, their data, or biological material

Policy information about studies with human participants or human data. See also policy information about sex, gender (identity/presentation), and sexual orientation and race, ethnicity and racism.

Reporting on sex and gender	n/a
Reporting on race, ethnicity, or other socially relevant groupings	n/a
Population characteristics	We obtained fresh frozen tissue from AApoA-I patients carrying G26R, L90P and R173P variants (n=3).
Recruitment	Specimens were obtained from the laboratory of late Dr. Merrill D. Benson at the University of Indiana.
Ethics oversight	The Office of the Human Research Protection Program granted exemption from Internal Review Board review because all specimens were anonymized.

Note that full information on the approval of the study protocol must also be provided in the manuscript.

Field-specific reporting

Please select the one below that is the best fit for your research. If you are not sure, read the appropriate sections before making your selection.

[x] Life sciences [ ] Behavioural & social sciences [ ] Ecological, evolutionary & environmental sciences

For a reference copy of the document with all sections, see nature.com/documents/nr-reporting-summary-flat.pdf

Life sciences study design

All studies must disclose on these points even when the disclosure is negative.

Sample size	We collected ~12,000 movies (except for the R173P kidney sample (~5000 movies)). The data collection parameters were documented in supplementary table 3.
Data exclusions	Particles with poor SNR each data-set in 2D classification were excluded from the final 3D reconstructions.
Replication	We performed multiple reconstructions with the dataset and observed the same results, thus confirming the reproducibility of these results.
Randomization	For the current design of the study, we analyzed all the samples based on their availability.
Blinding	Particles (segments) were picked by training and autopicking with TOPAZ to avoid bias in particle picking. For the downstream analysis, we used softwares (Relion 4.0) to avoid any potential bias.

Reporting for specific materials, systems and methods

We require information from authors about some types of materials, experimental systems and methods used in many studies. Here, indicate whether each material, system or method listed is relevant to your study. If you are not sure if a list item applies to your research, read the appropriate section before selecting a response.

Materials & experimental systems

n/a	Involved in the study	
x		Antibodies
x		Eukaryotic cell lines
x		Palaeontology and archaeology
x		Animals and other organisms
x		Clinical data
x		Dual use research of concern
x		Plants

Methods

n/a	Involved in the study	
x		ChIP-seq
x		Flow cytometry
x		MRI-based neuroimaging

Plants

Seed stocks

Report on the source of all seed stocks or other plant material used. If applicable, state the seed stock centre and catalogue number. If plant specimens were collected from the field, describe the collection location, date and sampling procedures.

Novel plant genotypes

Describe the methods by which all novel plant genotypes were produced. This includes those generated by transgenic approaches, gene editing, chemical/radiation-based mutagenesis and hybridization. For transgenic lines, describe the transformation method, the number of independent lines analyzed and the generation upon which experiments were performed. For gene-edited lines, describe the editor used, the endogenous sequence targeted for editing, the targeting guide RNA sequence (if applicable) and how the editor was applied.

Authentication

Describe any authentication procedures for each seed stock used or novel genotype generated. Describe any experiments used to assess the effect of a mutation and, where applicable, how potential secondary effects (e.g. second site T-DNA insertions, mosiacism, off-target gene editing) were examined.
