# Supplementary material for: Cryo-EM reveals structural variability of apolipoprotein A-I amyloid fibrils across organs, mutations, and clinical presentations
Source: Nat Commun. 2026 Apr 17;17:5324. doi: 10.1038/s41467-026-72150-z (PMC13273174; doi:10.1038/s41467-026-72150-z)
Supplement: Supplementary file 3 — Transparent Peer Review File [file 41467_2026_72150_MOESM3_ESM.pdf]

nature portfolio

Peer Review File

Cryo-EM reveals structural variability of apolipoprotein A-I amyloid fibrils across organs, mutations, and clinical presentations

Corresponding Author: Dr Lorena Saelices Gomez

This file contains all reviewer reports in order by version, followed by all author rebuttals in order by version.

Version 0:

Reviewer comments:

Reviewer #1

(Remarks to the Author)
The authors have extracted fibrils from three AApoA-I patients with potentially pathogenic variants. It is always heartening to see studies on ex vivo fibrils rather than those generated in vitro using non-physiological methods.

I have no experience of cryo-EM, and so this may be a naïve query. Since the patients are all heterozygous for the mutations, and the fibril preparations contain both WT and variant proteins (P4, L25), does this mean that the cryo-EM data were generated on mixed WT/variant fibrils rather than the "pure" variant fibrils? If so, does this affect the reconstruction, modelling and any conclusions that may be drawn from the data? Similarly, the fibrils contain N-terminal fragments, believed to be fibrillogenic drivers, and one might assume that their presence could materially affect fibril structure and thus data interpretation. Perhaps the authors could comment.

It appears from Fig S3A that the 173P heart fibrils contains only the WT peptide (173R) whilst the kidney sample contains only the variant (173P). Is this correct? If so, would you not expect fibrils from these tissues to exhibit different morphologies and structures? Does this finding give any insight into tissue targeting (WT vs. variant)?

The methodology for extraction/characterisation of fibrils is sound. I have no problem accepting the identification of ApoA-I or fragments in the fibrils. I would prefer to see some raw data, coupled with explanatory text, presented in the Supplementary Information rather than just having results stated as fact. This could include annotated spectra for the variant and wild type peptides, and representative ion chromatograms and spectra (raw and deconvoluted) for the N-terminal fragments. This information is important since the interpretation of the cryo-EM data is predicated on the correct identification of variants in the fibrils and their fragments.

Some specific points on the proteomics/MS:
• Is it possible to estimate the proportion of WT/variant protein in the fibril preparations, or that of N-terminal fragments to full length protein?
• Were all the ApoA-I variants (present in the Uniprot database) used in your searches, or just the expected variants, G26R, L90P and R173P? There is always the worry that search engines combine a strong set of peptide signals from the parent protein with a much weaker, possibly mis-identified, "variant" peptide. Are you happy with the number of variant peptide matches and the quality of the spectra? Showing that the retention times of the variant peptide is invariant across the three patient samples (e.g for G26R) can also increase confidence. A good test of your variant search approach would be to re-analyse the data using a set of variants that you know are absent; you might be surprised by the results.
• Were there any other non-ApoA-I proteins associated with the fibril preparations? You might expect some ATTR in the cardiac samples from 60+ year olds. When you obtained the smear for proteomics analysis, was the sample run into the gel for a short time (selecting for most proteins + oligomers), or for a much longer time where any smaller, non-fibril proteins may be lost?
• The mass acquisition range should be stated. Some of the tryptic peptides are very small (e.g. 24-27, DSGR and 24-26, variant DSR) and may not be acquired. Presumably these small peptides were identified through missed cleavages or semi-tryptic fragments. How confident are you in these assignments?
• Whilst Patient R173P (liver) fibrils shows the expected tryptic cleavage at 94K (residues 1-94, Fig S3A), the coverage of

the G26R (all), L90P (heart) and R173P (heart, kidney) fibrils only shows residues 1-93 (cleavage after 93V). Would this not imply that these fibrils contain mainly N-terminal fragments (such as 1-93) rather than the full sequence? Similarly, in the L60P patient there is coverage from 154-172, missing the normal tryptic cleavage product at 173R. Could this be another fibril fragment, or perhaps an artefact arising from the use of less robust semi-tryptic searches?
• How efficient was guanidine treatment in breaking up the fibrils for intact protein MW determination? Were you left with monomers that could be resolved by LC, or a poorly resolved mixture of monomers/oligomers? Did you identify full length ApoA-I as well as the N-terminal fragments, and were any other fragments identified? Working with fibrils can be difficult, and I would suspect that a 20um POROS R1 may lack the resolving power to separate the fragments. It would be understandable if the quality of the ion chromatograms and individual spectra were less than optimal.
• Please give the type of collagenase used.

Graham W Taylor

Reviewer #2

(Remarks to the Author)
The manuscript by Nguyen et al. addresses a critical gap in our understanding of hereditary apolipoprotein A-I amyloidosis (AApoA-I), which is a devastating rare disease where protein misfolding leads to organ failure and death.

The authors studied specimens from 3 patients who carry different variants of AApoA-I, and extracted from different organs. They collected 5 cryo-EM datasets, from which 3 fibril structures could be determined (variants L90P and R173P).
In 2 datasets (of the variant G26R) the fibrils were untwisted and their structure could not be solved.

Interestingly, the three fibril structures are all identical, even though they come from different patients who carry different mutations and from different organs. Given these fibril structures, it is quite clear that the G26R variant is not compatible with this fibril fold, as the Arg would sterically not fit and therefore must form a different fibril structure (which apparently is untwisted).

The resolution of the cryo-EM density maps is impressive, the cryo-EM work appears to be state-of-the-art. The resolution even allows for identifying a cis-conformation of Pro66, which is well confirmed by the provided density maps. Overall, the manuscript is clearly written, the figures are clear and informative. I only see two issues that should be addressed:

- The PDB deposited/provided density maps have a pixel size of 0.932 Å/px, which as explained in the Methods has been calibrated, but in the Supplementary Table 2 it still says 0.954 Å/px.

- The applied symmetry is C1 with a twist of -0.68º and a rise of 4.85 Å. However, looking at the map/model, the structure seems to have a 21-screw symmetry, i.e. I would have expected to see a twist of about 179.66º and a rise of 2.425 Å.

Version 1:

Reviewer comments:

Reviewer #1

(Remarks to the Author)
None

Reviewer #2

(Remarks to the Author)
I only had a few comments about the original manuscript which have been adequately addressed in the revised version of the manuscript. I do not see any further issues.

Open Access This Peer Review File is licensed under a Creative Commons Attribution 4.0 International License, which permits use, sharing, adaptation, distribution and reproduction in any medium or format, as long as you give appropriate credit to the original author(s) and the source, provide a link to the Creative Commons license, and indicate if changes were made.
In cases where reviewers are anonymous, credit should be given to 'Anonymous Referee' and the source.
The images or other third party material in this Peer Review File are included in the article's Creative Commons license, unless indicated otherwise in a credit line to the material. If material is not included in the article's Creative Commons license and your intended use is not permitted by statutory regulation or exceeds the permitted use, you will need to obtain permission directly from the copyright holder.
To view a copy of this license, visit https://creativecommons.org/licenses/by/4.0/

Responses to reviewers' comments

We sincerely thank the reviewers for their careful evaluation of our manuscript, and for the positive and insightful comments. We greatly appreciate the reviewer's recognition of the significance of our work in advancing the understanding of hereditary apolipoprotein A-I amyloidosis. Their constructive feedback has been extremely helpful in further strengthening the manuscript. We have carefully addressed all comments and believe that the revisions have improved the overall rigor, clarity, and completeness of the study. Below, we provide detailed responses to each point raised by the reviewers.

Reviewer #1 (Remarks to the Author):

Comment 1. Since the patients are all heterozygous for the mutations, and the fibril preparations contain both WT and variant proteins (P4, L25), does this mean that the cryo-EM data were generated on mixed WT/variant fibrils rather than the "pure" variant fibrils? If so, does this affect the reconstruction, modelling and any conclusions that may be drawn from the data? Similarly, the fibrils contain N-terminal fragments, believed to be fibrillogenic drivers, and one might assume that their presence could materially affect fibril structure and thus data interpretation. Perhaps the authors could comment.

Response: We appreciate the reviewer's concern. As the patients are heterozygous, we indeed observed the presence of both wild-type and variant tryptic fragments by mass spectrometry (Supplementary Figure 3), therefore indicating that the extracted fibrils contain a mixture of both variant and wild-type proteins. However, in the cases of L90P and R173P, the heterozygosity is unlikely to affect the structure and/or the model reconstruction, as the resolved fibril cores comprise residues 14 to 73, and both mutation sites lie outside this region (residue 90 and 173). Additional evidence supporting that mutations outside the fibril core do not influence our model reconstruction is that the fibril cores of L90P and R173P are identical.

As for the G26R variant, this concern is valid. Although we were unable to reconstruct the fibril core of G26R due to the untwisted morphology of the fibrils, we observed morphological heterogeneity in the 2D class averages. This morphological heterogeneity could be stemmed from differential contributions from both variant and wild-type species. The heterogeneity of the 2D classes could also arise from different Z-rotational views of the same fibril type.

We revised the text and added a sentence addressing this point as follows: "*This fibril core, spanning residues 14 to 73, appears unaffected by mutations outside this region. In contrast, the G26R substitution would introduce a bulky, ionic residue directly into a hydrophobic pocket, likely incompatible with the proper folding of this fibril core and perhaps resulting in a distinct untwisted fibril morphology (Fig. 2b). Additionally, G26R*

fibrils displayed morphological heterogeneity (Fig 2a, b). Given that the G26R patient was heterozygous, this variability may arise from different contributions of wild-type and variant molecules. Similar structural incompatibility may occur with other mutations located within the fibril core, such as W50R, L60R, L64P, and F71Y, due to potential steric clashes or interference with key hydrophobic interactions (Fig. S11)."

Comment 2. It appears from Fig S3A that the 173P heart fibrils contains only the WT peptide (173R) whilst the kidney sample contains only the variant (173P). Is this correct? If so, would you not expect fibrils from these tissues to exhibit different morphologies and structures? Does this finding give any insight into tissue targeting (WT vs. variant)?

Response: We thank the reviewer for this observation. As shown in Fig. S3A, it is correct that we could detect only the WT peptide (R173) in R173P heart fibrils, and only the variant peptide (P173) in the kidney sample. It is worth noting that detection of the peptide spanning residue 173 was inconsistent across all tissues and variants (G26R, L90P, and R173P) using trypsin digestion. For R173P, switching to Glu-C enabled detection of the variant peptide, but only in the kidney sample.

Given these inconsistencies across enzymes and tissues, we believe this variability arises from limitations in the mass spectrometry methodology rather than from tissue differences. Factors such as incomplete proteolytic digestion of the fibrils, low ionization efficiency, or suboptimal peptide length after enzymatic cleavage can impair detection. In this case, the presence of multiple adjacent arginine residues (R171, R173, R176) likely produces very short tryptic fragments that fall below reliable MS detection thresholds.

As noted in our response to Comment 1, the R173P mutation lies outside the fibril core; therefore, we do not expect different fibril morphologies in R173P (and L90P) samples.

Comment 3. The methodology for extraction/characterisation of fibrils is sound. I have no problem accepting the identification of ApoA-I or fragments in the fibrils. I would prefer to see some raw data, coupled with explanatory text, presented in the Supplementary Information rather than just having results stated as fact. This could include annotated spectra for the variant and wild type peptides, and representative ion chromatograms and spectra (raw and deconvoluted) for the N-terminal fragments. This information is important since the interpretation of the cryo-EM data is predicated on the correct identification of variants in the fibrils and their fragments.

Response: We appreciate the reviewer's suggestion. We have now included an additional figure (Supplementary Figure 4) showing the deconvoluted intact mass spectra of fibrils extracted from tissue samples of G26R, L90P and R173P patients, as a complement to Supplementary figure S3b. In addition, we have added a table (Supplementary Table 2) summarizing selected tryptic and semi-tryptic fragments detected in all the fibril samples

from tissues of all three patients showing the sequence coverage of ApoA-I as a complement to Supplementary Figure S3a.

The revised text for the passage "Fibril extraction and protein identification by mass spectrometry" now reads: "*Tryptic analysis revealed ApoA-I sequence coverage ranging from 71 to 89% across all fibril extracts (Fig. S3a and Table S.2). Using intact MS, we identified a fragment spanning residues 1 to 81 as the predominant species across all tissues and patients (Fig. S3b and Fig. S4). Additional patient-specific fragments were also identified: fragments spanning residues 1-67 and 1-71 in all G26R samples, 1-85 and 1-93 in all L90P samples, and 1-85 in all R173P samples (Fig. S4).*"

Some specific points on the proteomics/MS:

Comment 4. Is it possible to estimate the proportion of WT/variant protein in the fibril preparations, or that of N-terminal fragments to full length protein?

Response: We appreciate the reviewer's suggestion. Using our current mass spectrometry data (Supplementary Table 2), we were able to estimate the relative proportion of WT/variant protein only for the G26R sample, as comparable fragment pairs were not consistently detected for both L90P and R173P. In G26R, where the mutation lies within the fibril core and is therefore most likely to influence fibril folding, the estimated WT/variant ratio is approximately 55/45 (Supplementary Table 2).

Comment 5. Were all the ApoA-I variants (present in the Uniprot database) used in your searches, or just the expected variants, G26R, L90P and R173P? There is always the worry that search engines combine a strong set of peptide signals from the parent protein with a much weaker, possibly mis-identified, "variant" peptide. Are you happy with the number of variant peptide matches and the quality of the spectra? Showing that the retention times of the variant peptide is invariant across the three patient samples (e.g for G26R) can also increase confidence. A good test of your variant search approach would be to re-analyse the data using a set of variants that you know are absent; you might be surprised by the results.

Response: We appreciate the reviewer's careful consideration regarding potential false-positive variant identifications. We re-analyzed the data using a larger number of variant sequences as listed in UniProt, and using the same settings as the original semi-tryptic search. The results were identical to our original searches with no additional variant peptides identified. We are now satisfied with both the number of variant peptides matches and the quality of the spectra. We also updated the data analysis portion of the method to include that a GluC semi-specific search was used for the R173P mutant samples.

The revised method now read: *"Raw MS data files were analyzed using Proteome Discoverer v3.0 SP1 (Thermo), with peptide identification performed using a semitryptic*

(or semi-Glu-C for the R173P mutant sample) search with Sequest HT against the human reviewed protein database from UniProt along with the G26R, l90P, and R173P ApoA-I mutant sequences."

Comment 6: Were there any other non-ApoA-I proteins associated with the fibril preparations? You might expect some ATTR in the cardiac samples from 60+ year olds. When you obtained the smear for proteomics analysis, was the sample run into the gel for a short time (selecting for most proteins + oligomers), or for a much longer time where any smaller, non-fibril proteins may be lost?

Response: We appreciate the reviewer's concern. Indeed, a variety of non–ApoA-I proteins were detected in the mass spectrometry data. However, we are uncertain whether these proteins are directly associated with the AApoA-I fibrils or are residual tissue components. It is difficult to completely purify patient-derived fibrils from surrounding extracellular material. Some of these proteins may also be bound to collagen and collagen removal can release additional associated proteins into the final eluate. Given these uncertainties and the focus of this manuscript, we prefer not to overinterpret their presence.

The revised mass spectrometry result now read: "*Tryptic analysis revealed ApoA-I sequence coverage ranging from 71 to 89% across all fibril extracts (Fig. S3a and Table S.2), together with the detection of established amyloid signature proteins such as serum amyloid-P component, apolipoprotein E and apolipoprotein A-IV*.".

Comment 7: The mass acquisition range should be stated. Some of the tryptic peptides are very small (e.g. 24-27, DSGR and 24-26, variant DSR) and may not be acquired. Presumably these small peptides were identified through missed cleavages or semi-tryptic fragments. How confident are you in these assignments?

Response: We appreciate the reviewer's concern. We have now specified the mass acquisition range in the revised Methods section (please see below). The smallest peptides reliably identified in our searches were seven amino acids in length and the reviewer is correct that some amino acids were identified only in peptides with missed cleavages. To ensure confidence in these assignments, we applied stringent filtering criteria, including a high number of peptide spectrum matches (PSMs) and Xcorr values greater than 3.0. Based on these criteria, we are now confident that these peptide identifications are robust and not false positives.

The revised method now read: "*The mass spectrometer operated in positive ion mode with a source voltage of 2.5 kV and an ion transfer tube temperature of 300 °C. MS scans were acquired at 120,000 resolution in the Orbitrap with a scan range of m/z = 350-1700, and up to 20 MS/MS spectra were obtained in the ion trap for each full spectrum acquired using higher-energy collisional dissociation (HCD) for ions with charges 2-8.*"

Comment 8. Whilst Patient R173P (liver) fibrils shows the expected tryptic cleavage at 94K (residues 1-94, Fig S3A), the coverage of the G26R (all), L90P (heart) and R173P (heart, kidney) fibrils only shows residues 1-93 (cleavage after 93V). Would this not imply that these fibrils contain mainly N-terminal fragments (such as 1-93) rather than the full sequence? Similarly, in the L90P patient there is coverage from 154-172, missing the normal tryptic cleavage product at 173R. Could this be another fibril fragment, or perhaps an artefact arising from the use of less robust semi-tryptic searches?

Response: We thank the reviewer for this insightful observation. Based on multiple independent evidence presented in this paper, the fibrils indeed predominantly comprise of N-terminal fragments. These include: a substantially higher abundance of tryptic peptides derived from the N-terminal region (Supplementary Table 2); intact mass spectrometry showing dominant peaks corresponding to N-terminal fragments (Supplementary Figure 4); and cryo-EM densities (L90P and R173P variants) that are resolved for the N-terminal region.

Peptides detected from the C-terminal region, a possible indication of the presence of full-length ApoA-I, were detected at much lower abundance (Supplementary Table 2). This may reflect the partial proteolytic processing of the C-terminus during or after fibril formation.

Regarding the absence of the tryptic cleavage product at R173, this is most likely due to the nearby presence of multiple arginine residues (R171, R173 and R177), which would generate shorted peptides (less than 7 residues peptides) upon tryptic digestion. Such small peptides all below the reliable detection threshold of our mass spectrometry. Therefore, we do not attribute this observation to another fibril fragment, but rather to limitations of tryptic digestion methodology. For this reason, we employed GluC digestion for the R173P samples, which enabled the recovery of cleavage products at R173.

We revised the Results and Methods for this session as follows:

In the Results: "*Tryptic digestion was used in most cases; however, GluC digestion was employed to detect the R173P mutation, as tryptic cleavage around the mutation generated peptide fragments that are below the reliable MS detection threshold.*"

In the Methods: "*For identification of AApoA-1 R173P mutation, Glu-C enzyme (Promega) was used in addition to trypsin, with no other steps in the protocol altered, as tryptic digestion in the R171-177 region generated peptide fragments below the reliable MS detection range.*"

Comment 9. How efficient was guanidine treatment in breaking up the fibrils for intact protein MW determination? Were you left with monomers that could be resolved by LC, or a poorly resolved mixture of monomers/oligomers? Did you identify full length ApoA-I as well as the N-

terminal fragments, and were any other fragments identified? Working with fibrils can be difficult, and I would suspect that a 20um POROS R1 may lack the resolving power to separate the fragments. It would be understandable if the quality of the ion chromatograms and individual spectra were less than optimal.

Response: We appreciate the reviewer's insightful comments regarding the challenges of working with amyloid fibrils. In our experience, guanidine treatment only partially disrupts AApoA-I fibrils. We believe that the resulting material comprises a mixture of residual fibrils, monomeric species, and higher molecular weight species such as oligomers. Different species in the mixture is reflected by smeared bands on SDS–PAGE rather than discrete ones, as illustrated in the below figure. In this example, the R173P heart fibril sample was boiled at 100°C for 35 min, yet no clear band corresponding to full-length ApoA-I was observed.

We agree that, given the heterogeneous nature of the treated samples the resolving power of a 20 µm POROS R1 column is limited.

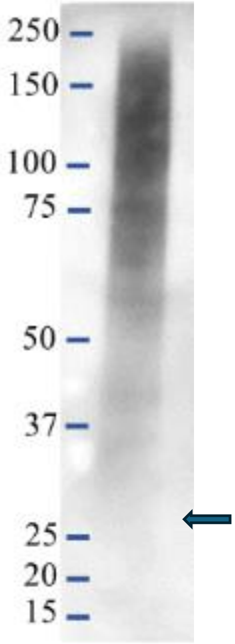

ApoAI monomer size ~ 30kDa

The revised method for MS sample preparation now read: "*The GnHCl treatment only partially disrupted AApoA-I fibrils, resulting in heterogeneous mixtures of residual fibrils, oligomers and monomeric species, which limited chromatographic resolution and precluded reliable detection of intact full-length ApoA-I.*"

Comment 10. Please give the type of collagenase used.

Thank you for this request. We have now specified the type of collagenase used in this study in the Methods section. The revised text now reads: "*After washing, the pellet was resuspended in 0.5 mL of 5 mg/mL collagenase solution (Clostridium histolyticum collagenase type IV stock prepared in Tris-calcium buffer*)".

Reviewer #2 (Remarks to the Author):

Comment 1. The PDB deposited/provided density maps have a pixel size of 0.932 Å/px, which as explained in the Methods has been calibrated, but in the Supplementary Table 2 it still says 0.954 Å/px.

Response: We thank the reviewer for pointing out this inconsistency. We have now changed the pixel size information in the Supplementary Table 2 to "*Pixel size (Å/px) (Original/Calibrated) 0.954/0.932*".

Comment 2. The applied symmetry is C1 with a twist of -0.68º and a rise of 4.85 Å. However, looking at the map/model, the structure seems to have a 21-screw symmetry, i.e. I would have expected to see a twist of about 179.66º and a rise of 2.425 Å.

Response: We appreciate the reviewer's insightful comment. We did attempt to impose a 2_1 screw symmetry during the final stages of 3D refinement. However, enforcing this symmetry worsened the map quality and resulted in a lower map resolution compared to the C1 3D refinement. Based on these comparisons, we therefore retained the C1 symmetry for the final reconstruction, as it provided the most reliable map for structural analysis and model building. Multiple rounds of CTF refinement, 3D classification (without image alignment), auto-refinement, and post-processing were performed sequentially to reach the highest possible resolution.

Now the text reads "*Throughout reconstruction, C1 symmetry was applied. Although the fibril architecture suggested a possible 2_1 screw symmetry, imposing this symmetry during the final refinement iterations resulted in a deterioration of map quality and reduced resolution. Therefore, C1 symmetry was retained for the final reconstruction to maximize map quality and reliability for structural interpretation*."
